# Atypically high influence of subcortical activity on primary sensory regions in autism

**DOI:** 10.1016/j.nicl.2021.102839

**Published:** 2021-10-05

**Authors:** Luigi Lorenzini, Guido van Wingen, Leonardo Cerliani

**Affiliations:** a Dept. of Psychiatry, Amsterdam UMC, University of Amsterdam, Amsterdam Neuroscience, Meibergdreef 5, 1105AZ Amsterdam, The Netherlands; b Dept. Radiology and Nuclear Medicine, Amsterdam UMC, VU University, Amsterdam Neuroscience, De Boelelaan 1117, 1081HV Amsterdam, The Netherlands; c Amsterdam Brain and Cognition, University of Amsterdam, Nieuwe Achtergracht 129-B, 1018WT, University of Amsterdam, The Netherlands; d Netherlands Institute for Neuroscience, Social Brain Lab, Meibergdreef 47, 1105BA Amsterdam, The Netherlands

**Keywords:** ABIDE, Autism Brain Imaging Data Exchange dataset, ASD, autism spectrum disorder, BMA, Bayesian model average, BMR, Bayesian model reduction, DCM, dynamic causal modelling, PEB, parametric empirical bayes, SRS, Social Responsiveness Scale, TD, typical development, MRS, Magnetic Resonance Spectroscopy, Autism Spectrum Disorder, Brain Connectivity, Dynamic Causal Modelling, Brain maturation, Primary sensory cortex, Subcortical nuclei

## Abstract

**Background:**

Hypersensitivity, stereotyped behaviors and attentional problems in autism spectrum disorder (ASD) are compatible with inefficient filtering of undesired or irrelevant sensory information at early stages of neural processing. This could stem from the persistent overconnectivity between primary sensory regions and deep brain nuclei in both children and adults with ASD – as reported by several previous studies – which could reflect a decreased or arrested maturation of brain connectivity. However, it has not yet been investigated whether this overconnectivity can be modelled as an excessive directional influence of subcortical brain activity on primary sensory cortical regions in ASD, with respect to age-matched typically developing (TD) individuals.

**Methods:**

To this aim, we used dynamic causal modelling to estimate (1) the directional influence of subcortical activity on cortical processing and (2) the functional segregation of primary sensory cortical regions from subcortical activity in 166 participants with ASD and 193 TD participants from the Autism Brain Imaging Data Exchange (ABIDE). We then specifically tested the hypothesis that the age-related changes of these indicators of brain connectivity would differ between the two groups.

**Results:**

We found that in TD participants age was significantly associated with decreased influence of subcortical activity on cortical processing, paralleled by an increased functional segregation of cortical sensory processing from subcortical activity. Instead these effects were highly reduced and mostly absent in ASD participants, suggesting a delayed or arrested development of the segregation between subcortical and cortical sensory processing in ASD.

**Conclusion:**

This atypical configuration of subcortico-cortical connectivity in ASD can result in an excessive amount of unprocessed sensory input relayed to the cortex, which is likely to impact cognitive functioning in everyday situations where it is beneficial to limit the influence of basic sensory information on cognitive processing, such as activities requiring focused attention or social interactions.

## Highlights

• The age-dependent decrease of subcortico-cortical connectivity is attenuated in ASD.
• Primary sensory regions remain less segregated from subcortical activity in ASD.
• This could underlie an excessive amount of sensory input relayed to the cortex.

## 1 Introduction

During the transition from childhood to adulthood our brain develops the ability to determine behavior on the basis of abstract representations and long-term plans, which are not primarily driven by current sensory stimuli, emotions and interoceptive feelings. This transition is reflected in the development of brain connectivity, and specifically in the increased independence of cortical information processing from subcortical inputs, coupled with the strengthening of long-range cortico-cortical connections within and between large-scale brain networks supporting higher-order distributed cognitive functions (Supekar et al., 2009).

In autism, the development of brain connectivity follows an atypical trajectory, and appears to be delayed or arrested at an immature stage. This is suggested by the persistent overconnectivity between cortical, subcortical and cerebellar regions (Di Martino et al., 2011, Cerliani et al., 2015, Woodward et al., 2017, Oldehinkel et al., 2019, Maximo and Kana, 2019), as well as by the underconnectivity between posterior and anterior brain regions within default mode, attentional and language networks (Herbert et al., 2003, Just et al., 2004, Müller et al., 2011, Kana et al., 2014). Such a connectivity pattern hampers the development of functional segregation between cortical networks (Holiga et al., 2019, Müller and Fishman, 2018), leading to an atypical functional integration among them (Hong et al., 2019; Rudie et al., 2012, Fishman et al., 2014). Specifically, recent neuroimaging studies suggest that functional integration in ASD is highly driven by current sensory information. In this respect, Hong and colleagues (Hong et al., 2019) showed that the functional segregation – measured as the order of stepwise functional connectivity (Sepulcre et al., 2012) – between primary sensory and transmodal cortices is significantly reduced in autism spectrum disorders (ASD). This situation might underlie an increase in the amount of basic sensory information reaching attentional and associative networks, and therefore the relevance of current sensory stimuli in determining behavior. A similar conclusion can be drawn from another study (Holiga et al., 2019) where decreased intrinsic functional connectivity within sensory and higher order fronto-parietal networks in ASD was associated with increased cross-talk between them. Finally, the presence of overconnectivity between subcortical and primary sensory regions (Cerliani et al., 2015, Woodward et al., 2017, Maximo and Kana, 2019, Ayub et al., 2021) suggests that deficits in filtering unwanted or irrelevant sensory stimuli in ASD might originate in early stages of sensory input processing, at the subcortical level.

**Fig. 1:**
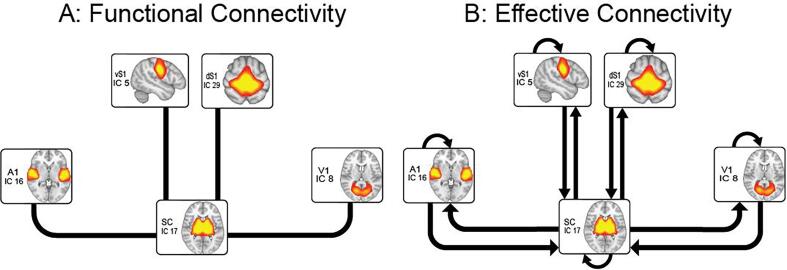
Functional and effective connectivity. A: Functional connectivity captures patterns of statistical dependence between regions of interest (ROIs) through the correlation of their fMRI time-series. Five ROIs, including subcortical nuclei (basal ganglia and thalamus) and primary sensory regions (dorsal and ventral somatosensory, primary auditory and primary visual cortex) showed increased functional connectivity in ASD compared to TD in our previous work (Cerliani et al. 2015). B: Effective connectivity models causal influences that one neural network exerts onto another. The figure depicts the connections we chose to model in our DCM analysis: bottom-up influence of subcortical nuclei on the primary sensory cortices; top-down influence of primary sensory regions on subcortical activity; and auto-connections, which in DCM model inhibitory connections of one neural system with itself (self-connections) and reflect its functional segregation – that is the sensitivity of a region to the influence of another modelled input (Zeidman et al., 2019a).

The reduced functional segregation of primary sensory regions, as well as the atypically high influence of subcortical regions over cortical processing, is compatible with an atypical developmental trajectory of brain connectivity in ASD, as several studies reported an age-related decrease in subcortico-cortical functional connectivity in typically developing participants but not in ASD (Iidaka et al., 2019, Cerliani et al., 2015). However these previous studies could not directly investigate the effect of age on the functional segregation of primary sensory regions and on the directional, bottom-up influence of subcortical over cortical regions, since the functional segregation and integration between regions was quantified using symmetric measures of functional connectivity – for instance the Pearson correlation coefficient – which do not yield a causal interpretation of brain dynamics, known as effective brain connectivity (Friston, 2011 – differences between functional and effective connectivity are illustrated in Fig. 1). Therefore, in the present study we used dynamic causal modelling ( Friston et al., 2003) to investigate the interaction between subcortical nuclei and primary sensory cortical regions in resting-state fMRI data. Importantly, we carried out the analyses using the recently developed spectral dynamic causal modelling (spDCM – Friston et al., 2014), which was specifically devised for resting-state fMRI data. We examined (1) the directional influence of subcortical activity on cortical sensory processing and (2) the intrinsic inhibition of each region, which reflects its sensitivity to the influence of other brain regions in the model (i.e. functional segregation), in both ASD and TD participants. We hypothesized age to be associated with a decrease in bottom-up connectivity and increased functional segregation of cortical regions in both ASD and TD participants. Crucially, however, we hypothesized that this age-dependent effect would be significantly attenuated in ASD with respect to TD participants.

## 2 Materials and methods

The code and the data used in this work are publicly available and can be found in the github repository: https://github.com/luislorenzini/ASD_DCM_subcortex_sensory.

### 2.1 Participants

We included in our study 166 participants with high-functioning ASD (all male, median age = 17.6, sd = 7.6) and 193 typically developing controls (TD – all male, median age = 16.9, sd = 6.6) sampled from the 1111 participants aggregated in the Autism Brain Imaging Data Exchange dataset (ABIDE I – Di Martino et al., 2014). The selection procedure (detailed in Fig. S1) ensured that (1) ASD and TD participants were matched by age, IQ, head motion and eye status in the scanner at the group level, (2) the images were devoid of problematic artifacts arising from image acquisition issues or motion. Table 1 reports the demographics of the final sample of 359 participants. Figs. S5 and S6 provide additional information on the distribution of participants’ age per site and across sites.

**Table 1:** Participants Demographics and Cognitive Scores.

	Mean (SD) [Range]	
	ASD (N = 166)	TD (N = 193)
Age, years	17.6 (7.6) [7–50]	16.9 (6.6) [6.5–39.4]
Full-scale IQ	109.6 (16.16) 71–148	111 (13.1) [73–146]
ADI-R Social (N = 93)	19.7(5.3)[7–28]	N/A
ADI-R Verbal (N = 94)	15.6(4.5)[2–25]	N/A
ADI-R Repetitive Behavior (N = 93)	5.8(2.58)[0–12]	N/A
ADOS Total (N = 171)	10.7(5.3)[0–22]	N/A
ADOS Communication (N = 170)	3.5(1.9)[0–8]	N/A
ADOS Social (N = 171)	7.1(3.8)[0–14]	N/A
ADOS Repetitive behavior (N = 142)	1.7(1.6)[0–8]	N/A
SRS (N_ASD_ = 111; N_TD_ = 108)	89.4 (32.4) [6–164]	22.2 (18.1) [0–103]

Abbreviations: ASD, autism spectrum disorder group; TD, typical development group; N/A, not applicable; ADI-R, Autism Diagnostic Interview – Revised ( Lord, Rutter, and Le Couteur 1994); ADOS, Autism Diagnostic Observation Schedule (Lord et al. 1989); SRS, Social Responsiveness Scale (Constantino et al., 2003, Constantino and Todd, 2003).

### 2.2 Regions of interest for the analysis of effective connectivity

We aimed to estimate the effective connectivity between subcortical nuclei and primary sensory regions during resting-state fMRI. To determine the location of these regions in a data-driven way, we carried out an independent component analysis (ICA) using FSL Melodic meta-ICA (Beckmann and Smith, 2004). All the details about data preprocessing and meta-ICA are described in detail in a previous study (Cerliani et al., 2015) and in the supplementary information. Notably, we strived to remove motion by (1) regressing the estimated motion parameters (2) carrying out ICA Aroma (Pruim et al., 2015) and (3) excluding participants featuring relatively high residual motion quantified by framewise displacement (mean framewise displacement across all time points > 0.34). Implementing meta-ICA (Biswal et al., 2010) allowed us to extract 19 spatially independent components mostly located in the gray matter, featuring high reproducibility across twenty-five subsets of participants and with high resemblance to functional networks recruited by task-based fMRI experiments (Smith et al., 2009, Laird et al., 2013). Our previous functional network connectivity analysis evidenced that among all components, the group of ASD participants showed a significantly higher interaction between one subcortical component – encompassing the basal ganglia and thalamus – and four primary sensory cortical networks – ventral and dorsal somatosensory, visual and auditory (Cerliani et al., 2015). Therefore in the present study we use the spatial maps associated with these 5 components (thresholded at Z > 3 from the meta-ICA results) to estimate differences in their functional segregation and directional interaction between ASD and TD participants using dynamic causal modelling (Karl J. Friston et al., 2014).

One of our anonymous reviewers raised the concern that given the relatively wide spatial extent of these regions of interest (ROIs), the ICA components could encompass heterogeneous subsets of voxels, while this risk could be reduced by using spheres of 6 mm centered around the local maxima of each ROI. In the present study it was important to use as ROIs the same used in our previous functional connectivity study (Cerliani et al., 2015), since we aimed at further investigating with DCM the nature of the increased subcortico-cortical interaction in ASD, which represented the main result of the previous study. In order to establish an unbiased relationship between the results of functional and effective connectivity, we were therefore bound to use the same regions of interest previously found using meta-ICA. However, we investigated in a supplementary analysis (see Supplementary Materials – “Choice of the region of interest: meta-ICA component vs. spherical ROI” and Fig. S3) whether using such spherical ROIs vs. the whole (thresholded) meta-ICA component would have decreased the heterogeneity within each ROI, but failed to find support for this possibility. This reinforces our confidence in the relative homogeneity of the regions of interest we used for DCM analysis.

### 2.3 Spectral dynamic causal modelling

Dynamic Causal Modelling (DCM – (K. J. Friston et al., 2003)) aims to model effective connectivity – that is the causal interactions between brain regions – in order to derive information about the direction and strength of each connection. While traditionally DCM was applied only to task-based fMRI, recently spectral dynamic causal modelling (spDCM) was specifically devised to estimate effective connectivity in resting-state fMRI data (Friston et al., 2014). spDCM uses a Bayesian framework to model directional interactions amongst brain regions based on their cross spectral densities, and obtain estimates of the strength of each connection. Technical details on spDCM can be found in the supplementary information and Fig. S4, as well as in the reference papers (Razi et al., 2015, Razi et al., 2017).

Spectral DCM was carried out using SPM 12 (https://www.fil.ion.ucl.ac.uk/spm) (Friston et al., 2007). Following well established procedures (Friston et al., 2016), in the first-level (single subject) analysis we only specified one full model per subject, including all the bottom-up (subcortico-cortical) and top down (cortico-subcortical) connections between our regions of interest, as well as the inhibitory self-connections within each region (Fig. 1B). Since direct connections between primary sensory cortices are anatomically implausible (Mesulam, 2000), cortico-cortical connections were not modelled, reducing the number of parameters to be estimated. Inversion (fitting) of the model to the data provided an estimation of single-subject DCM parameters, i.e. connection strengths. Comparison with reduced models was carried in the second-level (group) analysis (see below). Explained variance of full DCM models was inspected to ensure convergence. No subject was excluded due to poor data fit.

### 2.4 Parametric empirical Bayes

To test differences in DCM parameters at the group level, we used a recent implementation of SPM to model group effective connectivity in the context of DCM, known as Parametric Empirical Bayes (PEB) (Friston et al., 2016). In brief, this can be considered as a Bayesian second-level general linear model testing how subject measures (individual connection strengths) relate to the group mean and other group-level variables. This routine has the advantage of taking into consideration the full posterior density from the first level (single-subject) DCM to inform the second level results (Zhou et al., 2018).

The main PEB model included the mean, age, group and group-by-age interaction as between-subject variables of interest. To exemplify significant effects found in the interaction term, we specified a second model including the effect of age separately within the two groups. In each model, the rs-fMRI mean (across time points) framewise displacement (FD – Power et al., 2012) of each subject was also included as a nuisance variable, to model potential residual effects of movement. Finally, since the data of this ABIDE sample was collected in different sites, we also modelled site with additional dummy covariates (more on this below).

Following current standards (Karl J. Friston et al., 2016), Bayesian model reduction (BMR) was then used to estimate several nested (reduced) models by assuming one or more connections from the full model to be selectively switched off, and derived evidence directly from the full model. Bayesian model average (BMA) was subsequently employed to estimate a weighted average of the parameter strength based on nested models’ log evidence and estimate the influence of between subjects regressors. Further details on BMR and BMA procedures can be found in the supplementary information and Fig. S4.

### 2.5 Modelling site-related confounds

Data from the present ABIDE sample was acquired in 8 different sites. In order to model out this potential confound, we initially followed the standard procedure of introducing 7 dummy covariates (one less than the number of sites, to prevent rank deficiency of the model matrix) encoding each site as 1 for participants from that site, and 0 everywhere else. However, a preliminary analysis of variance revealed significant mean age difference across sites (F(7,351) = 22.07, p < 0.001, see Fig. S6), already highlighted in previous reports (Woodward et al., 2017). Therefore, such dummy variables prevent the age predictor from capturing the variance which is shared between age and site-related confounds, as in the general linear model only the variance in the dependent variable which is unique to a particular predictor is captured by that predictor. In other words introducing dummy variables to control for the effect of site – given the significant differences in age across sites – introduces (partial) collinearity in the model and effectively removes variability in DCM estimates (the mean for each site) which is explained by both age and the dummy variables used to model inter-sites differences, rather than by confounding differences between sites only. Such procedure can potentially make the results unstable and introduce false negatives in the estimation of the association between age and effective connectivity, which represents the main scope of our analyses. Additionally, one of our anonymous reviewers suggested us to assess the model after having orthogonalized the 7 dummy variables for sites with respect to the Age regressors (and subsequently to each other, to maintain their reciprocal orthogonality). We present the results in Figs. S9–S10 and anticipate that the results were virtually identical to those obtained without sites’ confounds. However, orthogonalization is a procedure that is usually discouraged because it is not devoid of limitations (Poldrack et al., 2011). For these reasons, we will report the results both with and without corrections for site, since it is not possible, given the data at hand, to separately model the variance in connectivity estimates which is due to either site-related confounds or to interesting differences in the age of the participants.

### 2.6 Interpreting PEB results

In the context of DCM, the probability of the effects predicted by the model is estimated in a Bayesian framework. Bayesian statistics incorporates prior knowledge of the event in the model to be tested – for instance the presence of a connection between two brain areas. Contrary to the frequentist approach, which does not explicitly test the probability of the hypothesized effect, in the Bayesian framework we test the probability of our specific hypothesis given the observed data. Therefore, the frequentist concept of statistical significance does not apply to Bayesian statistics which in turn provides information on the likelihood of the hypothesized effect. Here, the PEB outcome provides two types of information: 1) the estimated effect, which refers to the strength and the sign of the influence that each covariate exerts on each DCM connection, expressed in Hz: for example, an effect of age on the self-connection of S1 of + 0.14 means that the self-inhibition of S1 increases 0.14 times the age score; 2) the probability of the parameter, which represents the probability of the observed effect. Following previous DCM studies (Almgren et al., 2018), we will consider significant those experimental effects where the posterior probability of the DCM parameter given the data (P(M|Y)) exceeds 0.90, and therefore show a 90% confidence interval (hereafter CI) not including the zero (Makowski et al., 2019).

### 2.7 Association with symptoms severity

We then evaluated the relationship between effective connectivity and behavioural symptoms that are often observed in association with ASD, in a subsample of individuals for which Social Responsiveness Scale (SRS (Constantino et al., 2003)) scores are provided in the ABIDE dataset (n = 219). Linear mixed models were used to study the effect of SRS and its interaction with age on estimated DCM parameters (connectivity strengths) of regions showing significant association in PEB analysis. As for the PEB model, FD was added as a covariate. A random intercept was used to correct for the effect of the site. P-values were adjusted for multiple comparisons using false discovery rate (FDR). We chose to use linear models for easier interpretability of the results when interacting two continuous variables. However, for consistency with the DCM framework and to take into account first-level uncertainty, we also specified a PEB model including SRS, age and their interactions as independent variables. Mean FD was also used here to correct for motion and dummy variables for adjusting the effect of site.

## 3 Results

In our previous investigation on this sample from the ABIDE dataset (Cerliani et al., 2015) we reported that resting-state functional connectivity between subcortical and primary sensory regions was increased in ASD with respect to TD participants (Fig. S2). In the present study we hypothesized that this subcortico-cortical overconnectivity could be explained by an atypical development of bottom-up projections and functional segregation of the cortex. To test this hypothesis we examined the relationship between age and DCM parameter estimates for functional segregation (self-inhibition) and directional bottom-up influences. Group level statistics of estimated DCM connectivity parameters and explained variances are shown in Table S1 and Fig. S7.

### 3.1 Main effect of Age across all participants

**Fig. 2:**
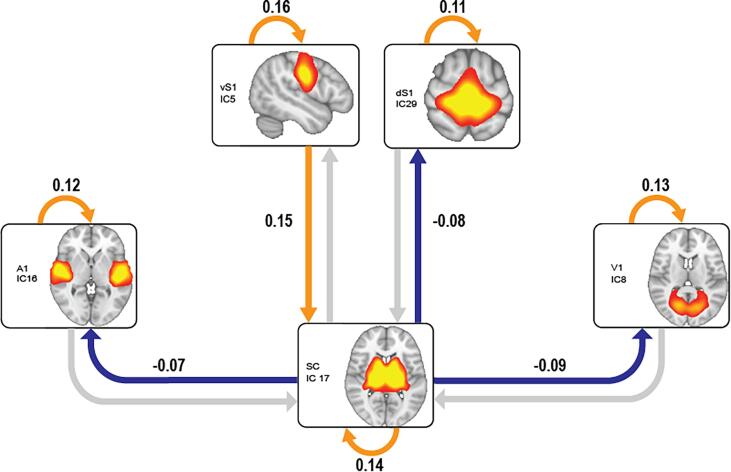
Main effect of Age. Association between Age and directed subcortico-cortical influence or regional functional segregation (self-connections) in the whole group. An increase in age results in decreased influence (in blue) of subcortical over cortical sensory activity, and increased segregation (in orange) of the intrinsic activity of each region from that of other regions in the model. In the case of the ventral somatosensory cortex, age is associated with an increased top-down influence (in orange) on subcortical activity. Orange and blue connections represent interactions showing a significant effect of age (90% CI on DCM parameter estimates outside zero). The DCM parameter estimate for each significant connection is reported close to the corresponding arrow. SPM graphical outputs and results of this analysis before and after site correction are reported in Fig. S9. (For interpretation of the references to colour in this figure legend, the reader is referred to the web version of this article.)

Fig. 2 shows the main effect of age on bottom-up and top-down influence between subcortical and cortical sensory regions in the whole sample of 359 participants (ASD + TD). In addition, it shows the effect of age on self-connections, which model each region’s excitatory-inhibitory balance: a stronger inhibitory self-connection reflects higher functional segregation of that region’s activity from the influence of the other regions in the model (Zeidman et al., 2019b).

Consistent with our predictions and previous literature, age was significantly associated with increasing functional segregation across all cortical and subcortical regions (self-connections in orange in Fig. 2). Age was also significantly associated with decreasing influence of subcortical activity on primary sensory regions across all sensory modalities (blue connections in Fig. 2). In the case of the ventral somatosensory cortex, age was significantly associated with an increasing top-down influence from the cortex on subcortical regions. Although our main focus was on the relationship between DCM estimates and Age, for completeness we also report the result of the valence of each connection independently from Age in the whole group (Fig. S8).

### 3.2 Reduced functional segregation of primary sensory regions in ASD

**Fig. 3:**
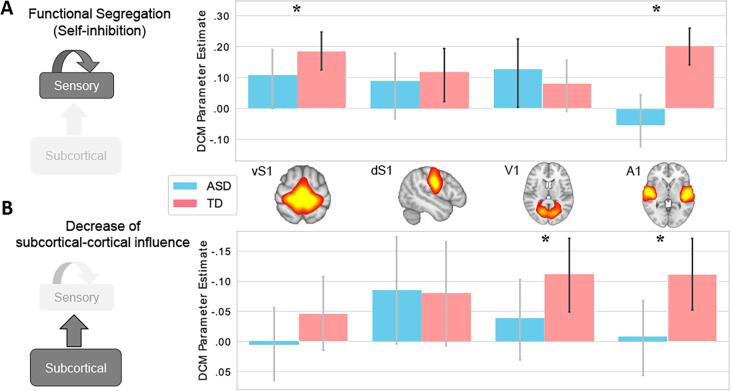
Age-related group differences in effective connectivity (blue = ASD, pink = TD). Error bars indicate the 90% CI around the DCM parameter estimates for the within-group DCM, in which age was modelled separately for ASD and TD participants. In DCM, a 90% CI not including zero is used to determine if the parameter estimates are to be considered significant (Almgren et al. 2018). Black error bars reflect 90% CI not including zero, therefore indicating a significant effect of Age. Asterisks denote a significant (90% CI) age-by-group interaction (TD > ASD). A: Effect of age on functional segregation. In TD, age is significantly associated with an increase of the functional segregation of primary somatosensory (vS1, dS1) and auditory (A1) regions, while in ASD this is the case only for V1. The age-by-group interaction effect is significant in vS1 and A1. B: Effect of age on subcortico-cortical influence: In TD, age is significantly associated with a reduced influence of subcortical regions on cortical sensory processing in the visual (V1) and auditory (A1) modalities (note that the values on the Y axis are inverted). By contrast, age is not significantly associated with changes of bottom-up influence in ASD participants. The age-by-group interaction confirmed the presence of a significant difference between ASD and TD in the reduction of bottom-up connectivity for these sensory modalities. SPM graphical outputs and results of this analysis before and after site correction are reported in Fig. S10. (For interpretation of the references to colour in this figure legend, the reader is referred to the web version of this article.)

Fig. 3A shows the DCM parameter estimates of functional segregation (self-connection) for each sensory region (and the associated 90% CI) when age was modelled separately in TD and ASD participants. The functional segregation of primary somatosensory (vS1, dS1) and auditory (A1) regions significantly increased with age in TD participants (black CI bars in Fig. 3A) while in ASD participants this effect was significant only in the visual modality (V1). When these parameter estimates were compared between groups – in a single model including both ASD and TD – the age-by-group interaction confirmed that age has a smaller effect on the increase of cortical functional segregation in ASD in the somatosensory (vSI) and auditory modality (A1) (asterisks in Fig. 3A).

This result shows that some primary sensory regions are more susceptible to be influenced by subcortical activity in ASD participants than in TD participants of comparable age. Conversely, age appeared to contribute to a higher functional segregation of the subcortical regions in ASD than in TD (not presented in Fig. 3), but this effect only showed a trend towards significance (P(M|Y) = 0.88).

Importantly, these and the following results (next paragraph) were significant only in relation to the age of the participants, while no main effect of group was significant if age was treated as a confound.

### 3.3 Persistent subcortical influence on cortical sensory processing

Fig. 3B shows the DCM parameter estimates for the influence of subcortical on primary sensory brain activity (and the associated 90% CI) when age was modelled separately in TD and ASD participants. Note that since we are showing the effect of age on the *decrease* in the influence of subcortical nuclei on primary sensory regions, we inverted the values on the Y axis. In TD participants, the subcortical influence on visual (V1) and auditory (A1) cortical activity significantly decreased with age, while this was not observed for any sensory modality in ASD participants. The age-by-group interaction confirmed that age was significantly associated with a stronger decrease of subcortical influence over visual and auditory primary cortical regions in TD than in ASD (Fig. 3B: asterisks).

SPM graphical outputs of the described analysis are reported in Figs. S9 and S10. Adding site into the PEB model did not change the direction of the effect and showed minimal reduction of posterior probabilities, though leading to non-significance for several nodes.

### 3.4 Association with Social Responsiveness Scale

**Fig. 4:**
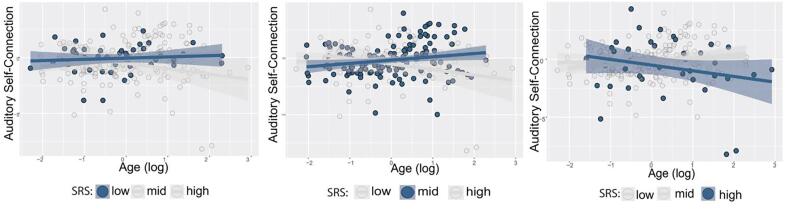
Effect of the interaction between age and SRS on DCM parameters. For clarity of presentation, participants’ SRS are stratified in low (total SRS < -1SD), mid (total SRS between −1SD and +1SD) and high (total SRS > 1 SD). Each panel shows the relationship between age and the self-connection strength in the auditory cortex for the three groups. Contrary to what we observed in low and mid SRS individuals, high symptom severity was associated with decreased A1 self-connection strength with age. Abbreviations: *SRS = Social Responsiveness Scale*.

In order to examine the association between neuroimaging results and behaviour, we studied whether similar effective connectivity alterations could be observed in relation with total SRS score. The interaction between age and SRS showed a trend to significant effect only on auditory cortex self-connectivity (p-value = 0.053 after q(FDR) = 0.05 correction). Specifically, the functional segregation of A1 decreased with age in participants with severe symptoms, while it remained stable or even increased in participants with mild or moderate behavioural symptoms (Fig. 4). Models’ coefficients are shown in Table S2. To further confirm these results using the DCM framework, we used PEB to study the effect of the interaction between SRS and age, hence taking into account the full posterior density of the first level DCMs. PEB results showed a significant effect of the SRS by age interaction term on A1 self-connection (P(M|Y) > 0.95), with high SRS and older age being associated with lower self-inhibition in this region (Fig. S11).

## 4 Discussion

### 4.1 Reduced functional segregation of primary sensory regions in ASD

The establishment of a relative functional segregation between cortical and subcortical brain processing represents a crucial step in the development of distributed, functionally specialized cortico-cortical networks which characterize the architecture of the mature brain (Supekar et al., 2009). This architecture allows the brain to preserve an equilibrium between local and distributed information processing, that is between functional segregation and functional integration (Caspers et al., 2013). The importance of such equilibrium is apparent during tasks which require both specialized processing and fast-paced integration of sensory stimuli, abstract concepts, and interoceptive information. Focused attention, language comprehension/production and social interaction are examples of such demanding everyday situations which are typically affected in autism.

In our analysis we observed that the functional segregation of primary somatosensory and auditory regions significantly increases with age in TD participants, while this effect is highly reduced in ASD participants. Probably the most interesting aspect of this result is the fact that in DCM, functional segregation – reflected in the parameter estimates of a brain region’s self-inhibition – is explicitly modelled as the contribution of GABA-ergic inhibitory projections to the sensitivity of each region (Bastos et al., 2012): a region that features low self-inhibition is more susceptible to be influenced by the activity of the other regions in the model (Zeidman et al., 2019a). To our knowledge, the reduced segregation of primary sensory regions represents the first – albeit indirect – neuroimaging evidence of atypical development of the local brain connectivity mediated by inhibitory projections. At the same time it should be emphasized that while this association between intrinsic BOLD fluctuations and self inhibitory connections is supported by the explicit modelling of these GABA-ergic projections in the DCM model (Marreiros et al., 2008), the hypothesis of a direct link between BOLD activity and inhibitory projections remains tentative due to the intrinsic difficulties in determining to what extent the fMRI signal is determined by an (im)balance of excitatory and inhibitory activity (Logothetis, 2008).

The investigation of inhibitory connections within and between cortical regions is of particular importance in autism research as many studies have proposed that this neuropsychiatric condition is associated with atypical development of intracortical inhibitory interneurons (Marín, 2012, Magueresse and Corentin, and Hannah Monyer, 2013, Ferguson and Gao, 2018), resulting in an imbalance of local excitatory/inhibitory signalling (Rubenstein and Merzenich, 2003, Belmonte et al., 2004, Pretzsch et al., 2021). Importantly, local imbalance of excitatory/inhibitory projections affects not only local circuits, but also the development of long-range projections interconnecting large-scale networks (Menon, 2013) due to reduced synchrony in the activity of distant clusters of minicolumns (Belmonte, 2004, Courchesne and Pierce, 2005). This atypical connectional architecture is consistent with recent findings showing atypical development of whole brain functional segregation and integration in ASD, characterized by facilitated access of basic sensory information to higher-level cognitive processes (Hong et al., 2019, Martínez et al., 2020) and cross-talk between primary sensory and higher-order regions (Holiga et al., 2019, Martínez et al., 2020).

In the specific context of DCM, the lower intrinsic connectivity of the primary sensory regions reflects the smaller self-inhibition of these regions in ASD compared to TD participants. Since thalamic projections are predominantly glutamatergic (Salt, 2002), this situation could allow an excessive influence of the excitatory afferents from the thalamus on cortical sensory processing. Similarly for the basal ganglia the excessive connectivity with primary sensory regions could hamper the development of effective fronto-striatal circuits which are involved in filtering out irrelevant or undesired stimuli. This possibility is supported by several reports of impaired sensorimotor gating in people with ASD (McAlonan et al., 2000, Perry et al., 2007, Madsen et al., 2014).

### 4.2 Persistent high influence of subcortical activity on cortical primary sensory regions in ASD

In a previous study we reported that ASD participants showed increased functional connectivity between subcortical and primary sensory regions (Cerliani et al., 2015). This effect could reflect an atypically enhanced (1) influence of subcortical activity on cortical sensory processing (bottom-up); (2) top-down modulation of subcortical activity; or (3) both. Since feedforward (bottom-up) and feedback (top-down) neuronal activity are well characterized in terms of their neurophysiological signatures (Wang, 2010), discriminating among these three cases would be possible only by complementing our fMRI data with EEG/MEG recordings (a currently active area of research in DCM (K. J. Friston et al., 2019, Jafarian et al., 2020)). Therefore our results about the relationship between age and directional subcortico-cortical interaction – which represents the focus of our investigation – should be considered as reflecting bottom-up connectivity only within the interpretative context of the employed fMRI DCM model.

Given the prevalence of sensory symptoms in ASD, such as hyperreactivity to sensory stimulation and the presence of stereotyped and repetitive behaviour, as well as the recent evidence of an increased functional connectivity between sensory and transmodal cortical regions (Hong et al., 2019), we specifically tested the hypothesis that the enhanced functional connectivity between subcortical and cortical regions in ASD would reflect an increased directional influence of subcortical activity on cortical processing in primary sensory regions. Our DCM analysis revealed that while in TD participants the influence of subcortical regions on primary sensory cortical regions decreases with brain maturation, this effect is largely not present in ASD participants. This situation could engender an excessive influence of unprocessed or undesired sensory information on cortico-cortical networks, overriding higher-order cognitive processes in determining the relevance of different cognitive representations to generate behavior.

This hypothesis of an increased influence of subcortical on primary sensory regions in ASD highlights the central role of deep brain nuclei – and especially of the thalamus – in gating incoming sensory stimuli (Woodward et al., 2017, Ayub et al., 2021), and the consequences that can derive from atypical or altered thalamic gating. Atypical thalamic filtering of sensory stimuli in ASD might be due to an imbalance of inhibitory and excitatory signalling (see also section 4.1), which has been evidenced using *in vivo* MRS by altered levels of glutamate in the primary auditory cortex (Brown et al., 2013) – resulting in increased cortical excitability – and of GABA in the thalamus (in ASD males) (Fung et al., 2021), as well as in the primary auditory and motor cortex (Gaetz et al., 2014). On the basis of the analysed fMRI data alone, we cannot conclude that our findings are a direct reflection of an atypical thalamic gating mediated by neurotransmitter imbalance in autism. However the link between sensory atypicalities, decreased thalamic gating and excitatory/inhibitory imbalance is suggested by several studies beyond the specific research on autism: (1) a recent study (in neurotypical adults) aimed at disrupting thalamic gating within the cortico-striato-thalamo-cortical circuit found increased DCM effective connectivity between thalamus and the posterior cingulate cortex upon administration of lysergic acid diethylamide (Preller et al., 2019); (2) the same substance results in a reduction of prepulse inhibition (Halberstadt 2015), a measure of sensorimotor gating, which as previously mentioned (section 4.1) is impaired in autism and schizophrenia (Madsen et al., 2014). While the aetiology of the imbalance in excitatory/inhibitory signalling in autism is different from the mechanisms examined in these studies, these reports are relevant for autism research in that they show how an imbalance in thalamocortical excitatory/inhibitory connectivity results in atypical sensory experience and behaviour. Finally (3) an alteration of thalamic gating has also been proposed to underlie the thalamocortical overconnectivity in schizophrenia (Anticevic et al., 2014) – which recent reports show to have a higher comorbidity with ASD than what is expected in the neurotypical population (Chien et al., 2021, Zheng et al., 2018). Such thalamocortical overconnectivity also predicted symptoms severity, although the specific association with sensory symptoms in schizophrenia was not examined.

A complementary reading of our results involves also considering the limitation mentioned above with respect to the neuronal interpretation of the fMRI effect we report. The significantly smaller age-related decrease in bottom-up activity in ASD that we found may reflect a smaller attenuation of feedforward neuronal activity in the gamma band, as the fMRI BOLD signal was shown to positively correlate with and explain a substantial amount of variance in power of gamma band oscillations (Lachaux et al., 2007, Scheeringa et al., 2011, Samogin et al., 2020). This would also be compatible with previous electrophysiological studies which found increased network efficiency (Kitzbichler et al., 2015) and feedforward functional connectivity between primary and secondary somatosensory cortex (Khan et al., 2015) in ASD. At the same time, those studies also reported decreased resting-state mu-beta power (Khan et al., 2015) and reduced network efficiency (Kitzbichler et al., 2015) in the beta band, which characterizes top-down feedback neuronal signalling (see also O’Reilly et al., 2017 for a meta-analysis of EEG/MEG studies in ASD). However, fMRI is less sensitive to the power in the beta band (Scheeringa et al., 2011, Scheeringa and Fries, 2019). This leaves open the possibility – suggested by one of our reviewers – that the age-related attenuation of the bottom-up connectivity could be also due, at least in part, to a potentiation of feed-back, beta-band signalling (Khan et al., 2015), which has been reported to occur during the transition from adolescence to early adulthood (Marek et al., 2018) and could be reduced in ASD. While our data are unsuited to test this hypothesis, it certainly represents a very interesting avenue of research, which could help to interpret the sometimes diverging results of fMRI-based over- or under-connectivity between the same regions/networks in different ASD studies (Müller et al., 2011, Khan et al., 2015, O’Reilly et al., 2017).

### 4.3 Situating the atypical subcortico-cortical connectivity in ASD in the framework of the underconnectivity/overconnectivity hypothesis

While social and communicative deficits are central to the diagnosis of autism, the clinical literature has constantly remarked the importance of sensory symptoms in ASD, generally qualified as hyper- or hyporeactivity to sensory stimulation (Gomot et al., 2008, Marco et al., 2011, Cascio et al., 2012, Green et al., 2013, Green et al., 2015, Robertson and Baron-Cohen, 2017, Cascio et al., 2019). More specifically, sensory perception in ASD is characterized by enhanced perceptual processing and discrimination, which suggests an increased cognitive focus on local over global features (Mottron et al., 2006, Minshew and Williams, 2007, Robertson and Baron-Cohen, 2017). At the same time, studies investigating basic measures of sensitivity in static sensory stimuli in autism failed to show higher thresholds for detection or discrimination in ASD than in TD. This recently led to the hypothesis that rather than a general bias towards local features, atypical sensory processing in ASD would particularly manifest in the slower dynamic integration of perceptual information over space and time, possibly due to the increased amount of cognitive resources necessary to process noisy sensory information (Robertson and Baron-Cohen, 2017).

This hypothesis fits well in the framework of the underconnectivity/overconnectivity theory (Belmonte, 2004, Just et al., 2004), according to which the global architecture of brain connections in autism could be characterized by inefficient long-range connections, supporting functional integration across different cognitive domains, coupled with an excess of local connections. The presence of local overconnectivity, consistent with decreased levels of GABA-ergic signalling and reduced minicolumnar size, would yield both a local and a distal effect on the development of brain connectivity. Locally, it would elicit indiscriminately high regional activation for any incoming sensory signal, thereby decreasing the selectivity of salient over irrelevant environmental stimuli (signal vs. noise) (Belmonte, 2004). At the same time, increased local signalling would negatively impact the formation of long-distance projections, resulting in delayed and/or reduced top-down modulatory projections, further decreasing the selectivity among the current environmental sensory stimuli (Menon, 2013).

The directional subcortico-cortical overconnectivity in ASD we report here could reflect an excessive corticopetal flow of basic sensory information – although we remark that our methods are not capable of directly quantifying exchange of information. This would result in a decreased signal-to-noise ratio in primary cortical regions targeted by subcortical projections, due to the increased presence of irrelevant sensory stimuli (Belmonte et al., 2004). In turn, this would pose a challenge to attentional and higher-order cognitive networks in terms of fast-paced dynamic integration of the current sensory activity. Such increased connectivity is apparent in ASD not only between subcortical and primary sensory regions, but also between the latter and transmodal regions which are directly connected to saliency and attentional networks, as suggested by recent findings of local overconnectivity at the transition between primary and transmodal sensory regions, coupled with a delayed transition of functional connectivity to high-order regions in the fronto-parietal and default mode network (Hong et al., 2019).

Importantly, this evidence allows to reframe the idea of ‘local’ overconnectivity in terms of functional hierarchy of neural information processing (Mesulam, 2000, Sepulcre et al., 2012, Margulies et al., 2016, Tian et al., 2020): while the hypothesis of long-range underconnectivity in ASD is consistent with many findings of decreased anatomical and functional connectivity between frontal and parietal brain regions (Just et al., 2012), evidence supporting local overconnectivity has only in part received a topographical localization ( Rudie and Dapretto, 2013, Keown et al., 2013; Supekar et al., 2013). Indeed, while topographical proximity is generally a good predictor of anatomical or functional connectivity, the presence of distributed networks in the brain shows that distant regions can be more connected to each other than to regions which are topographically closer, but have different functional specialization. The same rationale applies also to the reciprocal connectivity between entire functional networks, which reflects the hierarchy of information processing in the brain (Margulies et al., 2016). Primary sensory and subcortical regions are topographically relatively distant, but they are monosynaptically connected with each other, and represent immediately subsequent steps in the information processing hierarchy. Our results showing higher connectivity between subcortical and primary sensory regions therefore supports the idea that local overconnectivity in ASD should be conceptualized, and investigated, both in terms of topographical and functional proximity.

### 4.4 Limitations

The use of solely cross-sectional samples represents a limitation to the current study which could hamper the interpretation of age-related effects and their interaction with the pathology. However, the ABIDE data mostly include only baseline measurement leaving no room for regressing out within-subject variability from our analyses. Another limitation of the study is the presence of a statistically significant association between age and site, which prevents the possibility of effectively correcting the PEB model for the effect of scanning sites without removing age-related variability in the DCM estimates. Therefore, when adding dummy variables for each site, some of the model connections failed to reach statistical significance (Fig. S9, S10). However, as previously mentioned in the Methods, given the significant differences in mean age across sites (Fig. S6), the site confounds correlate with differences in Age, and therefore do not represent appropriate predictors for the unique variability associated with confounding differences between sites. Moreover, when adding sites as orthogonal dummy variables to age, the results were similar to our first model. For this reason, we presented the results using both with and without dummy variables for sites, and with site orthogonalization. In this context, we showed that the site-corrected and uncorrected models are very comparable in terms of directionality of results and the main findings (Age-related self-connection and bottom-up connection of A1, as well as its relationship with Age) remain significant.

It is also important to mention that our results – as well as most of those considered in the studies referenced here – have been obtained under the assumption of the stationarity over time of connectivity patterns between regions. Recent studies have started to relax this assumption, and investigate the dynamic reconfiguration of fMRI-based connectivity patterns in ASD and TD (see for instance Rashid et al., 2018, Mash et al., 2019, Marshall et al., 2020– in our Supplementary Discussion Notes). By implementing a more in-depth examination of connectivity patterns over time, this approach – both in functional and in effective connectivity (Zarghami and Friston, 2020) – will be valuable to describe the specifics of the interplay between functional integration and functional segregation in ASD, and to help interpret the results of static functional connectivity, like those we obtained in our investigation.

## 5 Conclusion

In the present study we hypothesized that the directional influence of subcortical activity on primary sensory cortices would be increased in ASD with respect to TD participants of the same age range, thereby reducing the segregation of primary sensory regions in ASD. This would provide evidence to understand the nature of the previously reported subcortico-cortical overconnectivity in ASD (Di Martino et al., 2011, Cerliani et al., 2015, Woodward et al., 2017, Oldehinkel et al., 2019, Maximo and Kana, 2019).

To test this hypothesis we modelled the bottom-up effective connectivity from basal ganglia and thalamus to the primary sensory regions in a relatively large group of participants (N = 359). We found that (1) the influence of subcortical regions on primary visual and auditory cortices significantly decreased with age in TD, but not in ASD participants; (2) the functional segregation of somatosensory and auditory cortices from subcortical activity significantly increased with age only in TD participants, while this was the case only for the primary visual cortex in ASD participants.

These results suggests that the recently detected increased and ectopic interaction between primary sensory and higher order cortices in ASD (Hong et al., 2019, Holiga et al., 2019) originates already at the subcortical level, which is consistent with the decreased functional segregation of subcortical from cortical brain processes in ASD (Di Martino et al., 2011, Cerliani et al., 2015, Maximo and Kana, 2019). The evidence of a specific directionality in this high connectivity from subcortical to cortical regions brings support to the idea that such hyperconnectivity could represent one of the brain mechanisms causing hyperreactivity to sensory stimuli in ASD.

## 6 Ethics Approval and Consent to Participate

As reported in the original ABIDE paper (Di Martino et al., 2014), “All contributions were based on studies approved by the local Institutional Review Boards, and data were fully anonymized (removing all 18 HIPAA (Health Insurance Portability and Accountability)-protected health information identifiers, and face information from structural images). All data distributed were visually inspected before release.”

## 7 Availability of data and material

The data supporting the conclusion of this article are available in the Autism Brain Imaging Data Exchange (ABIDE), http://fcon_1000.projects.nitrc.org/indi/abide/;

## Funding

This work was supported by the Netherlands Organization for Scientific research (NWO/ZonMw Vidi 016.156.318)

## Author contributions

LC conceived and designed the research. LC and LL performed the analysis. LC, LL and GW interpreted the results. LL and LC drafted the manuscript. LL, LC and GW edited and revised the manuscript. All authors read and approved the final manuscript.

## CRediT authorship contribution statement

**Luigi Lorenzini:** Conceptualization, Methodology, Formal analysis, Writing – original draft, Writing – review & editing. **Guido van Wingen:** Conceptualization, Writing – review & editing, Supervision, Funding acquisition. **Leonardo Cerliani:** Conceptualization, Methodology, Formal analysis, Writing – original draft, Writing – review & editing, Supervision.

## Declaration of Competing Interest

The authors declare that they have no known competing financial interests or personal relationships that could have appeared to influence the work reported in this paper.
