# Supplementary material for: Atypically high influence of subcortical activity on primary sensory regions in autism
Source: Neuroimage Clin. 2021 Oct 5;32:102839. doi: 10.1016/j.nicl.2021.102839 (PMC8503568; doi:10.1016/j.nicl.2021.102839)
Supplement: Supplementary data 1 [file mmc1.docx]

Supplementary Information

# Sample selection


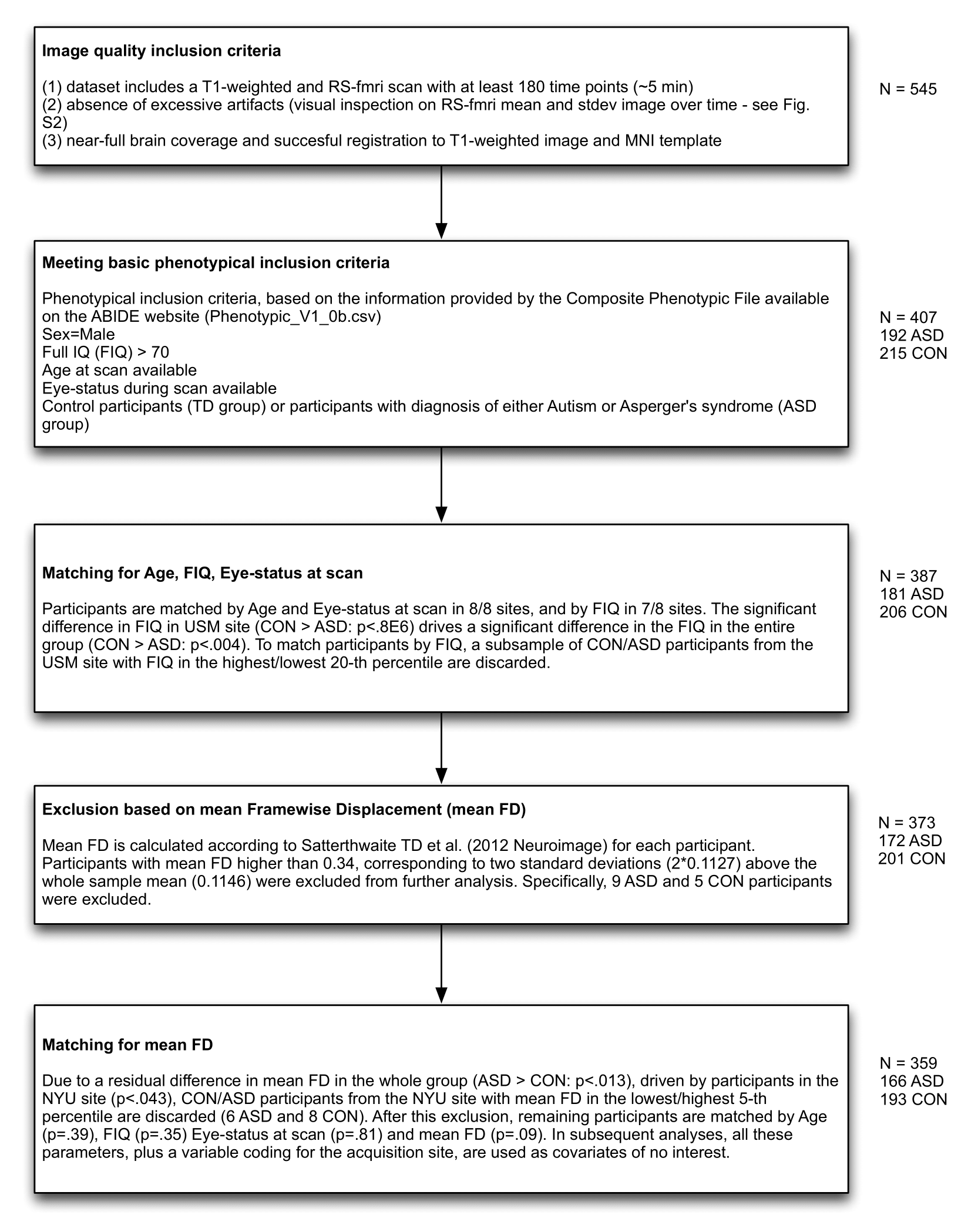


**Figure S1.** Pipeline used to derive the final sample of ASD and TD participants data used for the present study, as well as for our previous functional network connectivity study [(Cerliani et al. 2015)](https://paperpile.com/c/vtEbRx/QDSs).

# Preprocessing and selection of the regions of interest based on a previous functional connectivity study

All the preprocessing steps and the subsequent independent component analysis (ICA) - which was used to define the seed regions of interest for the present effective connectivity study - are described in detail in a previous study [(Cerliani et al. 2015)](https://paperpile.com/c/vtEbRx/QDSs).

To summarize, we used FSL to preprocess the resting-state fMRI images including brain extraction, linear motion correction, slice timing correction, spatial smoothing, and linear registration to the T1-weighted anatomical scan. To define the regions of interest which would then be used to extract the subject-level characteristic time courses to feed into the effective connectivity analysis, we used the results of a previous study of ours [(Cerliani et al. 2015)](https://paperpile.com/c/vtEbRx/QDSs) which employed independent component analysis (ICA) on the same subset of 359 participants from the ABIDE database [(Di Martino et al. 2013)](https://paperpile.com/c/vtEbRx/XIaRk). This choice was motivated by the fact that the results of that study provided the evidence of increased subcortico-cortical connectivity that represents the ground for our current hypotheses about the atypical directional influence of subcortical regions onto primary sensory regions in ASD which is currently tested using dynamic causal modelling [(Friston et al. 2014)](https://paperpile.com/c/vtEbRx/j4LOf).

ICA was carried out using FSL Melodic [(Beckmann and Smith 2004)](https://paperpile.com/c/vtEbRx/NNXS). The spatially independent components extracted by ICA represent the regions of interest used in the previous as well as in the present study.

To estimate robust spatial independent components, temporally concatenated melodic ICA was carried out 25 times on subsets of 112 participants randomly chosen from the dataset, and the final spatial components were obtained by a meta-ICA on the results of the first-level ICAs. Out of the resulting 52 automatically estimated meta-ICA components, we selected 19 of them mostly located in the gray matter, featuring high reproducibility across subsets, high resemblance to functional networks recruited by task-based fMRI experiments, temporal frequency spectrum in the low-frequency range of resting-state networks.

After thresholding the 19 spatial components at Z > 3 (Z values estimated by meta-ICA), their characteristic time course was extracted via dual regression [(Nickerson et al. 2017)](https://paperpile.com/c/vtEbRx/44eR4), bandpass filtered (0.009-0.08 Hz), and used to estimate functional connectivity between networks within each participant, with the aim of subsequently comparing these functional connectivity estimates between groups after regressing out the effect of age, IQ, eye status (open/closed) at scan, motion (mean framewise displacement) and site of acquisition (one dummy variable for each site - 1 to prevent rank deficiency)

Inference on the difference in functional connectivity between ASD and TD participants was carried out using permutation testing [(Nichols and Holmes 2002)](https://paperpile.com/c/vtEbRx/Qxr3y) with 20,000 permutations for each cell of the 19x19 functional connectivity matrix. The resulting p values (from a two-tailed test) were corrected for multiple comparison using false discovery rate [(Genovese, Lazar, and Nichols 2002)](https://paperpile.com/c/vtEbRx/1GjMn) (q(FDR)= 0.05) considering all the p values in the upper triangular connectivity matrix, that is 19x18/2 p values (identical to the values in the lower triangular as the functional connectivity matrix is symmetric). This resulted in a final corrected p threshold of 0.001. This analysis revealed that functional connectivity between one subcortical network - encompassing the basal ganglia and thalamus - and four primary sensory cortical networks - ventral and dorsal somatosensory, visual and auditory - was significantly higher in ASD than in TD. No other group-differences were significant after correction for multiple comparisons (Figure S2). These 5 networks were used in the present study as regions of interest (ROI) for dynamic causal modelling [(Friston et al. 2014)](https://paperpile.com/c/vtEbRx/j4LOf).

**
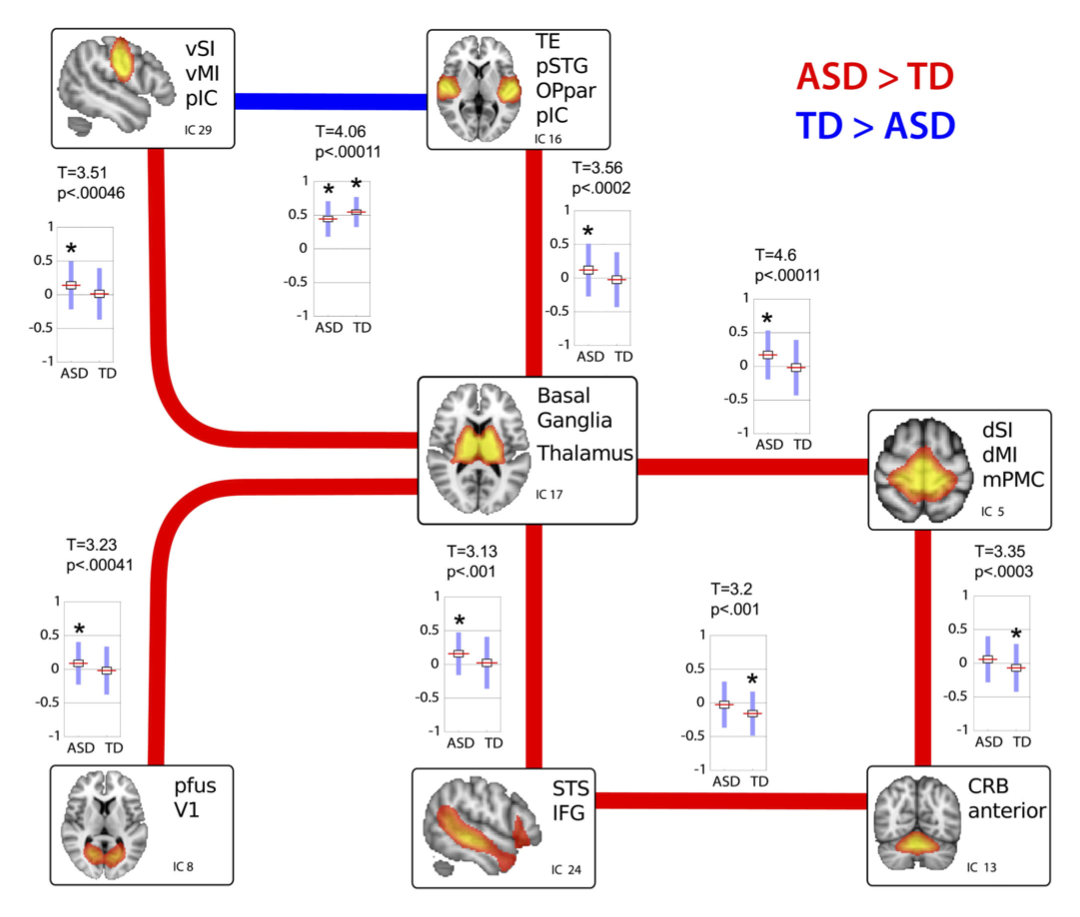
**

**Figure S2. Increased subcortico-cortical functional connectivity in ASD vs TD participants.** (representation of the data from Fig. 2 in [(Cerliani et al. 2015)](https://paperpile.com/c/vtEbRx/QDSs), available at <https://pubmed.ncbi.nlm.nih.gov/26061743/>): Group differences in functional network connectivity strength are shown as lines (red indicates increased functional network connectivity in autism spectrum disorder [ASD] with respect to typically developing [TD] participants, and blue indicates the reverse situation) together with boxplots of the Pearson correlation values within each group. Boxplots report the mean (red line), SEM (blue rectangle around the mean), and SD (whiskers) of group-level functional network connectivity values. Results were obtained by comparing the between-network functional connectivity of 166 ASD group and 193 TD group participants using nonparametric permutation testing (20,000 permutations) and correcting the final results with q[FDR] = 0.05, leading to a final threshold of P < .001. Asterisks indicate that the mean within-group functional connectivity is significantly different from zero. Converting the correlation scores to z scores using Fisher r to z transformation yielded almost identical results (eFigure 5 in the original study). Anatomical abbreviations: **IC29** : ventral primary somatosensory and primary motor cortex, posterior insular cortex; **IC16** : primary auditory cortex (TE), superior posterior temporal gyrus, parietal operculum, posterior insular cortex; **IC5** : dorsal primary somatosensory and primary motor cortex, medial premotor cortex; **IC13** : anterior dorsal cerebellum; **IC24** : superior temporal sulcus, inferior frontal gyrus; **IC8** : posterior fusiform gyrus, primary visual cortex.

# Choice of the region of interest: meta-ICA component vs. spherical ROI

In our study, we carried out an effective connectivity analysis with the specific aim to further the understanding of the atypical subcortico-cortical interaction found in our previous functional connectivity study [(Cerliani et al. 2015)](https://paperpile.com/c/vtEbRx/QDSs). Therefore, to establish an unbiased relationship between the results of functional and effective connectivity we estimated the eigenvariate for DCM analysis using the entire region estimated by the meta-ICA analysis (see all the details in the paragraph above), rather than choosing as region of interest a sphere of given radius, centered around the local maxima of the meta-ICA region.

One of our anonymous reviewers pointed out that given the wide spatial extent of the meta-ICA components, they might include subregions whose functional properties - as quantified by the fMRI signal - is reciprocally heterogeneous. In this situation the eigenvariate of each region would not adequately represent all of its voxels, but rather a mixture of the time courses representing different subregions. Such situations represent a common issue of every clustering problem, as the balance between homogeneity within and between clusters - in our case, the meta-ICA components - is at least in part determined by theoretical and methodological choices. The employed ICA algorithm [(Beckmann and Smith 2004)](https://paperpile.com/c/vtEbRx/NNXS) extracts spatially independent components whose voxels’ time course are more similar within than between components. Additionally, in our case, the reproducibility of a component across subsets of participants (together with proportion of gray matter it encompassed) represented an important methodological criterion to consider each component as a set of voxels with similar time course. The need to establish a link between functional and effective connectivity results represents the main theoretical reason why we choose to use these spatial components - as opposed to spheres around their maxima - to extract eigenvariates to be fed in the DCM analysis. Conversely, choosing spheres of a given radius around the local maxima of the components could have biased the selection of voxels towards those with the highest Z values at the expenses of those which show significant activity after thresholding (described in the paragraph above), making them not adequately representative of the entire set of voxels grouped by meta-ICA in one component. Specific problems of choosing spherical ROIs on the results of ICA are also that - for a relatively low order number - (1) most ICA components are bilateral (like the one we focus on in our study) and (2) spatially distributed across different brain regions (e.g. for the default mode, the saliency and the attentional networks), which would make it difficult to select one specific center for the spherical ROI.

Despite these justification for our methodological choices, we agreed it was interesting to follow the reviewer’s suggestion and carry out an additional analysis, aimed at quantifying the heterogeneity within the entire spatial component (hereafter: whole ROI) and within a region defined by a sphere of 1.5 times the voxels size (that is 6mm) in radius, centered on the local maxima of the whole region (hereafter: spherical ROI). Specifically we wanted to assess whether our choice of using the whole ROI could have resulted in an increase in the heterogeneity across the voxels within one ROI, compared to using localized spherical ROIs instead.

To this aim we carried out, for each subject and each of the 5 ROIs, a singular-value decomposition (SVD) of the time courses of all the voxels within a region. Practically this was implemented using the [principal component analysis function in Matlab](https://nl.mathworks.com/help/stats/pca.html), which internally uses SVD. We used SVD since this is the method employed to extract the eigenvariate of each region, to be fed into DCM analysis. Also, one advantage of SVD, over other algorithms that can be used for clustering, is that it does not require any additional input from the experimenter, besides the data matrix.

The SVD quantifies the amount of variance explained by the first and by the subsequent components, as a ratio of the total variance across all the voxels in a region. We first qualitatively estimated the decrease in explained variance in the first principal components (pc), and then quantified the difference between the variance explained by different pc’s between spherical and whole ROI for the first 10 components (the first 2-3 likely being more representative of common signal, rather than noise, across voxels).

A smooth - rather than steep - decrease of the explained variance between the first and the second components, for instance, would suggest the presence of two distinct, relatively homogeneous subregions inside the whole ROI. However, since - as described above - the homogeneity of a region/cluster is a characteristic which is difficult to quantify without a term of comparison, this suggestion would be reinforced only if the decrease in explained variance would be comparatively steeper in the spherical, more localized ROI.


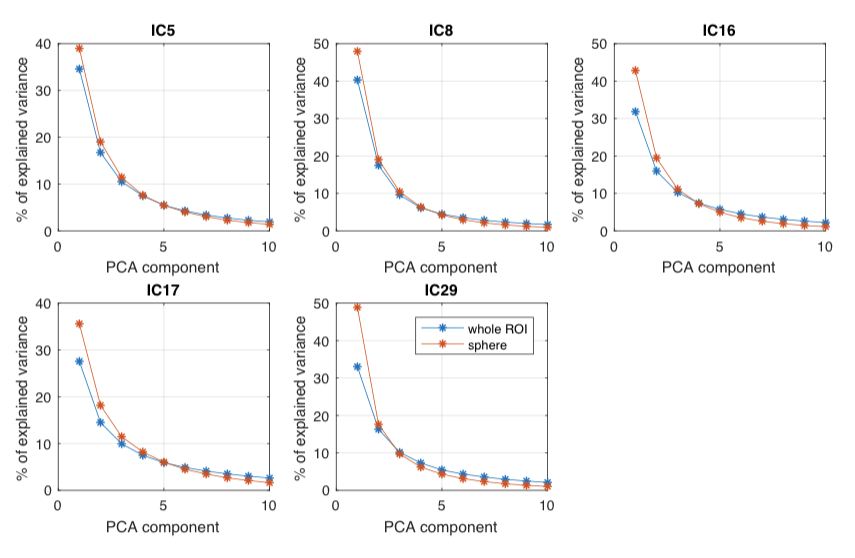


**Figure S3.** Mean percentage of variance (across 359 participants) explained by each component of the singular value decomposition across all the voxels encompassed by either a spherical ROI of 6mm radius (orange line) around the local maxima of the ICA components (thresholded Z > 3 ), or by the entire mask of the thresholded ICA component (blue line).

The figure above shows the descriptive results of this analysis: in all ROIs, a substantially higher amount of variance is explained by the first component with respect to the second, which represents a first qualitative indicator that the whole set of considered voxels in each ROI features a good degree of reciprocal homogeneity. More importantly, the steepness in the decrease of explained variance appears to be comparable between the whole and the spherical ROI, suggesting that if the whole ROI would contain separate subsets of voxels with homogeneous within-subset signal, the same description would fit the situation for the spherical ROI.

When we compared the mean amount of variance (across the 359 participants) explained by each component in either ROIs - using a paired t-test - we observed that the variance explained by the first component in the spherical ROI was significantly higher than in the whole ROI. This was expected, since we forced the selection of voxels only to the nearest neighbour of the local maxima, therefore to voxels with very similar time courses. As a side note, since all of our examined meta-ICA components were bilateral, a representative spherical ROI was determined by the union of the spherical ROIs centered around the maxima in the right and left hemisphere. Therefore, this result also shows the homogeneity of two anatomically distant regions within the same meta-ICA component.

More relevant for our purposes, when we compared the higher-order components - which would indicate heterogeneous subsets of voxels within the ROI - we failed to show that the variance explained by the second (or higher) component(s) was significantly higher in the whole than in the spherical ROI, which would have suggested more heterogeneity in the former than in the latter. Only in components higher than the 4th one - which arguably reflect more noise than signal - and only in some cases, the variance explained in either ROIs differed, but significantly never above a mere 1%, which is negligible considering the amount of variance explained by the first few components (~ 30 - 40% or more).

In summary, our choice of using the whole meta-ICA component to estimate the eigenvariates to feed into DCM was motivated mostly by the necessity to maintain a consistency between the regions of interest used in the previous functional connectivity analysis, and in the present effective connectivity analysis. However this further examination shows that potential concerns about the heterogeneity of the meta-ICA components, due to their spatial extent, could be raised also when choosing instead spherical ROIs centered around the local maxima. From another point of view, this analysis showed that a relative homogeneity within each ROI is achieved in both cases.

# Spectral dynamic causal modelling

To identify the effective connectivity between the 5 ICA-based spatial maps (IC17 - basal ganglia and thalamus; IC5 dorsal somatosensory; IC29 - ventral somatosensory; IC8 - primary visual; IC16 - primary auditory) we employed spectral dynamic causal modelling (spDCM) [(Friston et al. 2014)](https://paperpile.com/c/vtEbRx/j4LOf). This framework is of particular interest in modelling resting-state fMRI time-series due to the parameterization of endogenous fluctuations by characterizing them in terms of their cross-spectral densities. Moreover, compared to the previous version of DCM for resting state (i.e. stochastic DCM) this eliminates the need of estimating the neural fluctuations, speeding up and simplifying model inversion [(Razi et al. 2015)](https://paperpile.com/c/vtEbRx/gnHTz).

DCM was carried out using SPM 12. For each subject, the full model included all the bottom up (subcortico-cortical) and top down (cortico-subcortical) connections between our regions of interest, and the inhibitory self-connections within each region, by setting the prior variance to 1 allowing them to be informed by the data. Since direct connections between primary sensory cortices are anatomically implausible [(Mesulam 2000)](https://paperpile.com/c/vtEbRx/Vyizn), cortico-cortical connections were not modelled, setting the prior variance to zero, reducing the number of parameters to be estimated.

The model inversion for each subject provided the estimation of the connection strength parameters which best explained the observed data, resulting in the expected value and covariance (uncertainty) for each connection, as well as the free energy approximation to log model evidence, indicating the quality of each model in terms of accuracy and complexity.

#

# Parametric empirical Bayes

We subsequently tested differences in parameter strength due to between subject variability in the diagnosis, the age and the interaction between these two. To do so, we used a recent implementation of SPM to model group-level connectivity in the context of DCM, known as parametric empirical Bayes [(Friston et al. 2016)](https://paperpile.com/c/vtEbRx/49Cnn). This can be considered as a hierarchical Bayesian second-level general linear model that models how subject measures (individual connection strengths) relate to the group mean and other group-level variables. Unlike previous approaches to investigate group differences between DCM parameters which rested on classical inference approaches on individual expected values, this routine has the advantage of taking into consideration the full posterior density from the first level DCM, including both the estimated strength of each connection and the uncertainty (covariance), to inform the second level results [(Zhou et al. 2018)](https://paperpile.com/c/vtEbRx/yCEHV).

We therefore created a general linear model with 4 mean-centered variables of interest, i.e. the mean, the group, the age and the interaction between age and group, as the multiplication of age by group. We also included one nuisance variable with the mean framewise displacement (FD) for each subject in order to exclude potential effects of movement, allowing our PEB model to express the influence of our between-subject variables (each column of the design matrix) on the within-subject measures (parameter strength) as well as estimating the between-subject variability.

#

# Bayesian model reduction

After having inverted the individual full model and the group PEB model we used Bayesian model reduction and comparison to evaluate differences of effective connectivity across subjects. With this routine, several nested (reduced) PEB models are specified assuming one or more connections from the full model to be selectively switched off, and are then rapidly estimated deriving the model evidence directly from the full model. The Bayesian model average is subsequently used to estimate a weighted average of the parameter strength using the log evidence of the nested models [(Friston et al. 2016)](https://paperpile.com/c/vtEbRx/49Cnn).

Because we were interested in specific bottom-up or top-down connections, we constrained the Bayesian model reduction to 60 templates by selectively switching off just bottom-up or top-down connections.

To sum up, we first fitted a full model on the individual data obtaining the estimation of the parameters (connections) for each subject, we then brought this to the second (group) level using parametric empirical Bayes and finally we performed Bayesian model reduction and averaging. A schematic representation of the analysis pipeline is shown in Figure S4.


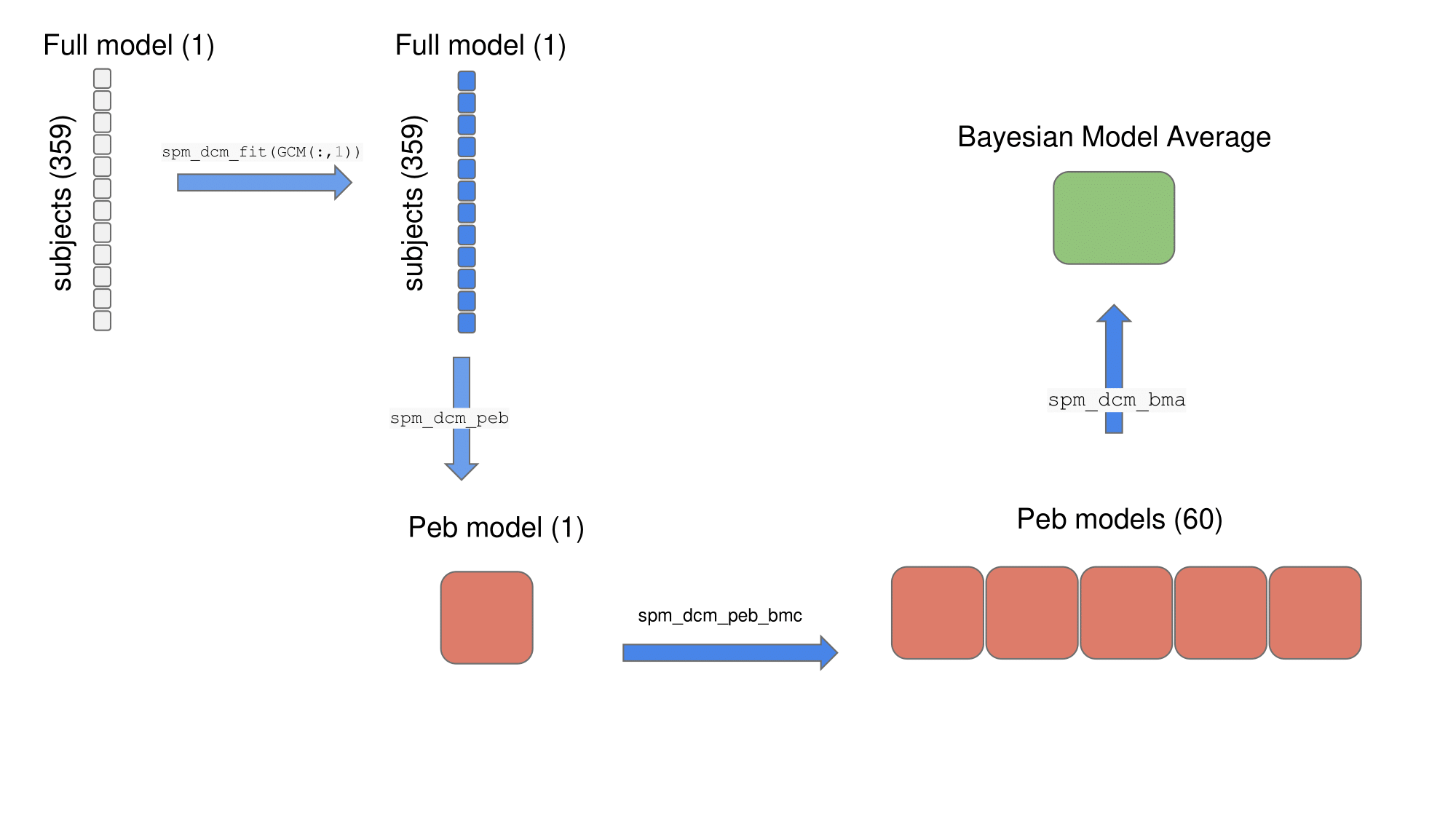


**Figure S4. A brief overview of the DCM pipeline** (adapted from [(Friston et al. 2016)](https://paperpile.com/c/vtEbRx/49Cnn)**.** The unfilled squares correspond to the specified models while the filled one to fitted models. Each column of the array corresponds to a particular model (with the full model on the first column). Each row corresponds to a particular subject. The figure illustrates how the analysis has been carried out. Firstly, we inverted the full model to the data obtaining the posterior probability and the parameter strengths. We then created a single PEB model (spm_dcm_peb) from the single subject full models and compared this with reduced PEB models (spm_dcm_peb_bmc). Finally we examined the BMA over the 60 PEB models.

# Supplementary Results

## Participants’ Age

Distribution of age across the whole sample included in the analysis is reported in Figure S5.


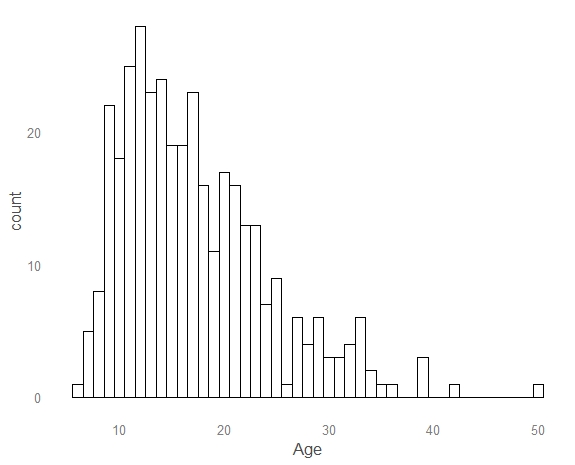


**Figure S5.** Age distribution across the whole sample of the included 359 participants.

As reported in the manuscript, using analysis of variance (ANOVA) we found differences in the mean age across sites significant (F(7,351) = 22.07, p < 0.001). Figure S6 illustrates age distribution across the 8 sites.


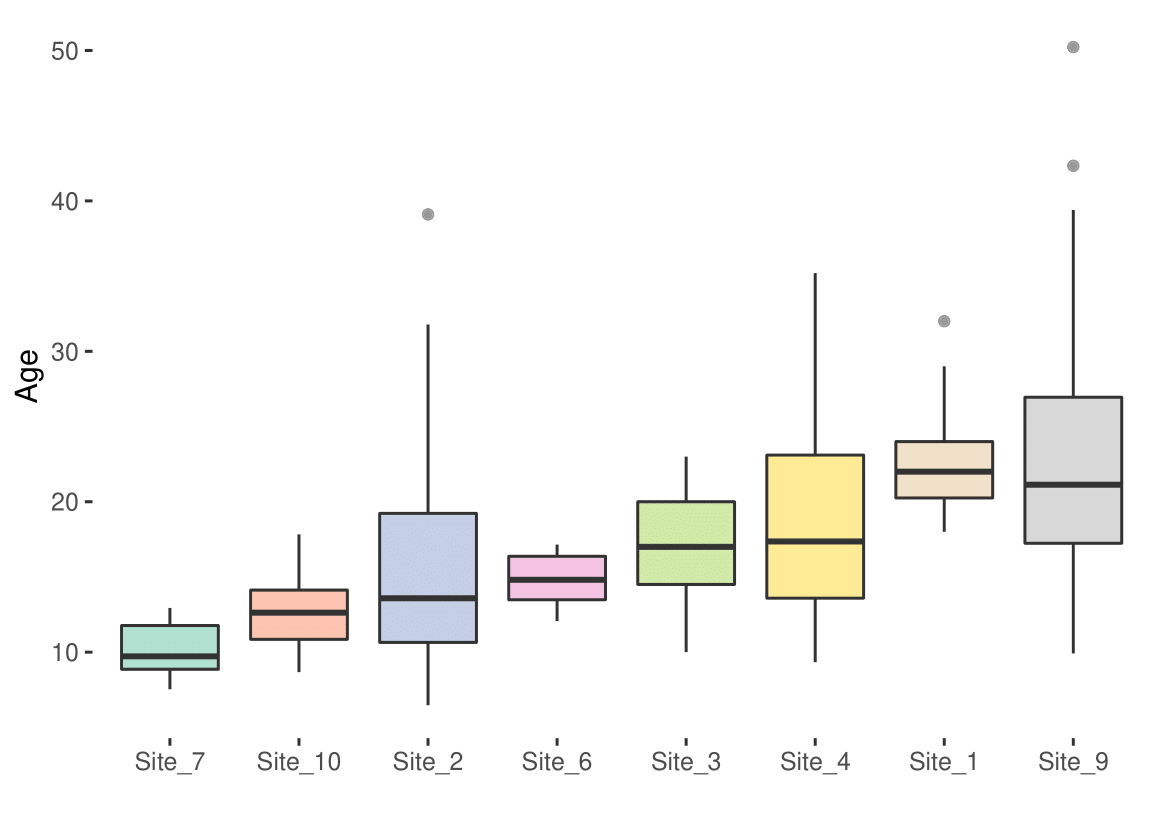


**Figure S6. Mean age differences across sites.** Differences in age between sites, ordered from the lowest to the highest age per site.

## Connectivity Parameters Statististics

**Table S1. Connectivity parameters group statistics**

| Connection | Posterior Expectations (mean ± SD) | Posterior Probabilities (mean ± SD) | Posterior Covariance  (group mean) | Valence |
| --- | --- | --- | --- | --- |
| Subcortical  -Subcortical | 1.79 ± 3.25 | 0.94 ± 0.11 | 0.003 | - |
| Subcortical-vS1 | -0.13 ± 2.0 | 0.95 ± 0.12 | 0.002 | - |
| Subcortical-dS1 | 0.15 ± 1.3 | 0.95 ± 0.13 | 0.002 | + |
| Subcortical-V1 | -0.11 ± 1.5 | 0.95 ± 0.12 | 0.002 | - |
| Subcortical-A1 | 0.07 ± 1.1 | 0.95 ± 0.13 | 0.001 | + |
| vS1-Subcortical | 0.07 ± 2.0 | 0.93 ±0.14 | 0.001 | + |
| vS1-vS1 | 0.24 ± 2.0 | 0.97 ± 0.10 | 0.003 | - |
| dS1-Subcortical | 0.03 ± 1.6 | 0.93 ± 0.13 | 0.001 | + |
| dS1-dS1 | 0.05 ± 2.3 | 0.97 ± 0.08 | 0.003 | - |
| V1-Subcortical | -0.03 ± 1.9 | 0.93 ± 0.12 | 0.001 | - |
| V1-V1 | -0.20 ± 3.4 | 0.98 ± 0.08 | 0.004 | - |
| A1-Subcortical | 0.13 ± 1.5 | 0.92 ± 0.13 | 0.002 | _ |
| A1-A1 | 0.30 ± 2.2 | 0.97 ± 0.08 | 0.003 | - |

For each connection, the group mean and standard deviation (where applicable) of the DCM outputs are reported, including posterior expectations, posterior probabilities and posterior covariance. Abbreviations: vS1 = ventral primary somatosensory cortex; dS1 = dorsal primary somatosensory cortex; V1 = primary visual cortex; A1 = primary auditory cortex.

##

##
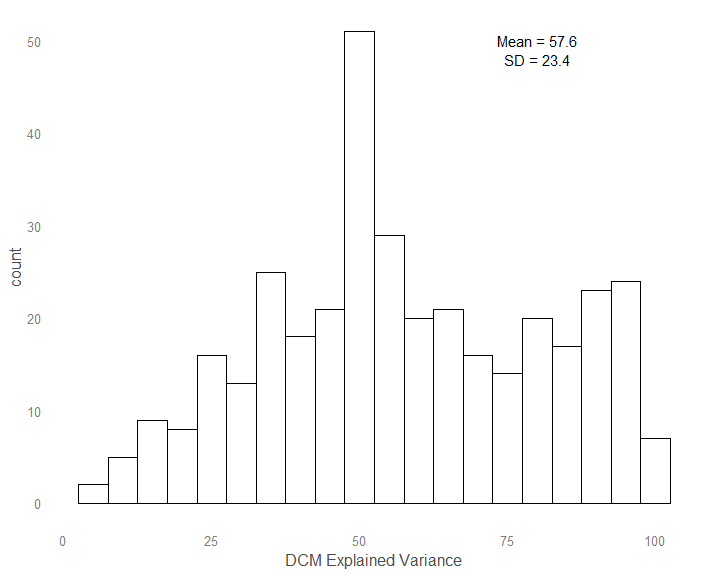


**Figure S7 DCM explained variance across the whole group.** The histogram shows the distribution of first-level (subject-level) DCM explained variance across the whole group (n = 359). *Abbreviations: DCM = Dynamic Causal Models; SD = Standard Deviation.*

##

## Mean Group Connectivity

PEB first estimated the baseline connectivity, i.e. the mean effective connectivity across the whole group between the modelled regions. The effective connectivity matrix diagonal (Figure S8) shows the estimated values for DCM inhibitory self-connections, which are unitless log scaling parameters of the default value of -0.5Hz [(Zeidman et al. 2019)](https://paperpile.com/c/vtEbRx/FBZac). Therefore, positive values stand for higher self-inhibition of the region, and vice-versa. Across the whole group, we found that the subcortical region had stronger self-inhibitory connections compared to primary sensory cortices. For the inter-region connections, we found excitatory influences of subcortical nuclei on dS1 and A1 and inhibitory influences on vS1 and V1. Top-down connections were mostly excitatory besides the influence except the connection between V1 and subcortical regions.


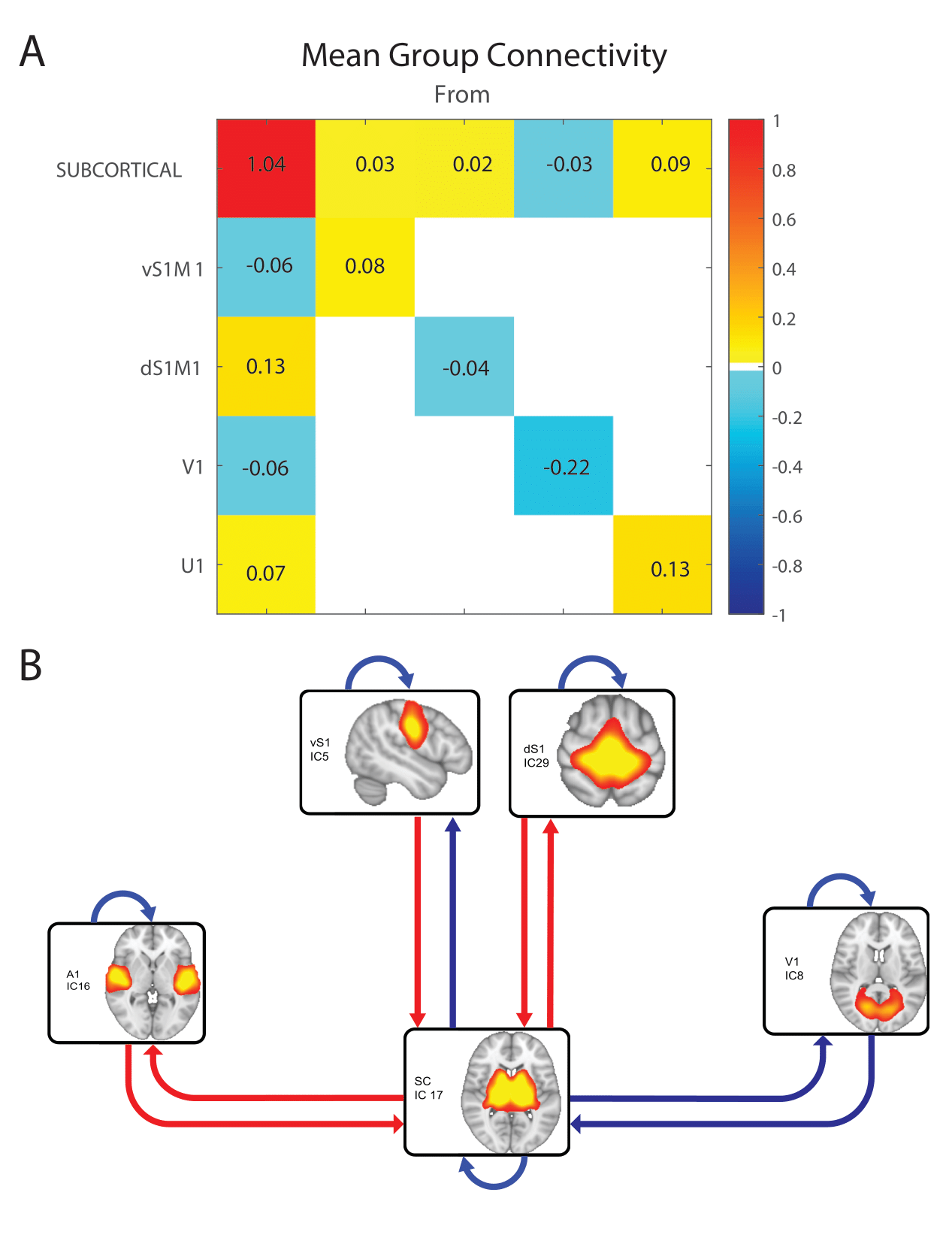


**Figure S8. Mean baseline effective connectivity across the whole group.** The PEB output when looking at the first (mean) specified variable. For bottom-up (values in the first column) and top-down (first row) connections, the colors and reported values represent the valence of the connection itself (red = excitatory; blue = inhibitory). Self-connections (main diagonal) are instead all modelled as inhibitory connections and the reported values are scaling parameters of the default value of -0.5Hz.

##

## Effect of age on network connectivity in the whole group

We found a main effect of age on DCM connections. As discussed in the text, intrinsic self-inhibition of both primary sensory and subcortical networks increased with age. This effect likely reflects the consolidation of the intrinsic circuitry within a functionally specialized network, and the progressive functional segregation between subcortical and cortical networks which occurs during development.

Moreover, age was generally associated with a decrease in bottom-up connections, reaching primary sensory cortices from subcortical nuclei and an increase of top-down feedback cortical afferents to the basal ganglia and thalamus. Figure S9 shows SPM graphical output of this relationship and the effect of correcting for site. However, as mentioned in the main text and shown in the previous section, site was related with age and created therefore multicollinearity in the PEB model.


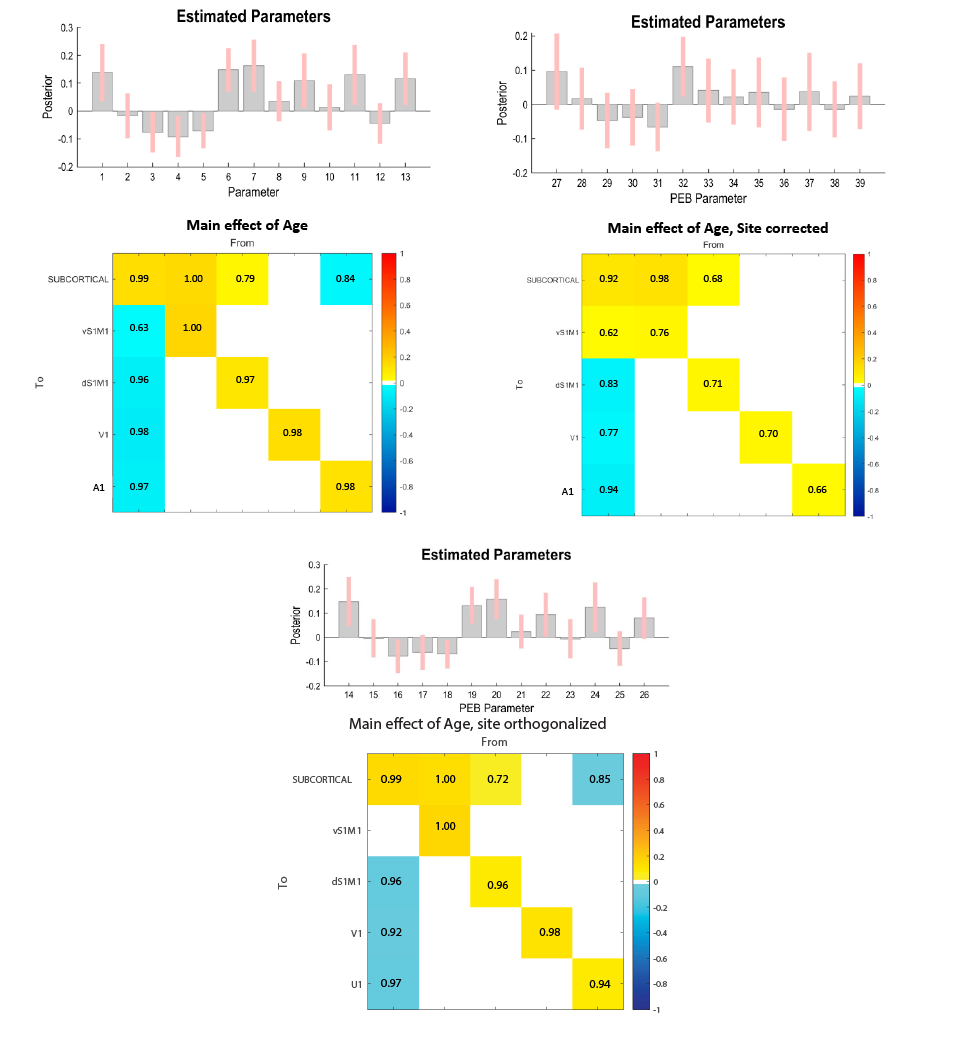


**Figure S9. Main effect of Age.** SPM graphical output when displaying the influence of age on DCM parameters. Top left: output of the analysis run without correcting for site; Top right : output of the analysis run correcting for site; Bottom row: output of the analysis run orthogonalizing site to age. For each result we show the barplot (*top*) showing effect sizes (gray bars) and 90% confidence intervals (pink lines), and the effective connectivity matrix (*bottom*) showing the main effect of age on intrinsic connectivity for each region (values on the diagonal), bottom-up (values in the first column) and top-down (first row) connections. Colors show estimated effect in Hz while annotated numbers show posterior probabilities for each connection.

## Effect of the interaction between age and group on network connectivity

Our main result was found looking at the effect of the interaction between age and group on DCM parameters. In brief, while TD showed a typical development of subcortico-cortical connections, with a weaker influence of basal ganglia and thalamus on primary sensory cortices, in ASD participants we found an attenuated functional segregation of sensory networks. Here we report the SPM graphical output of this analysis and the effect of adding site as a covariate in the PEB model (Figure S10).


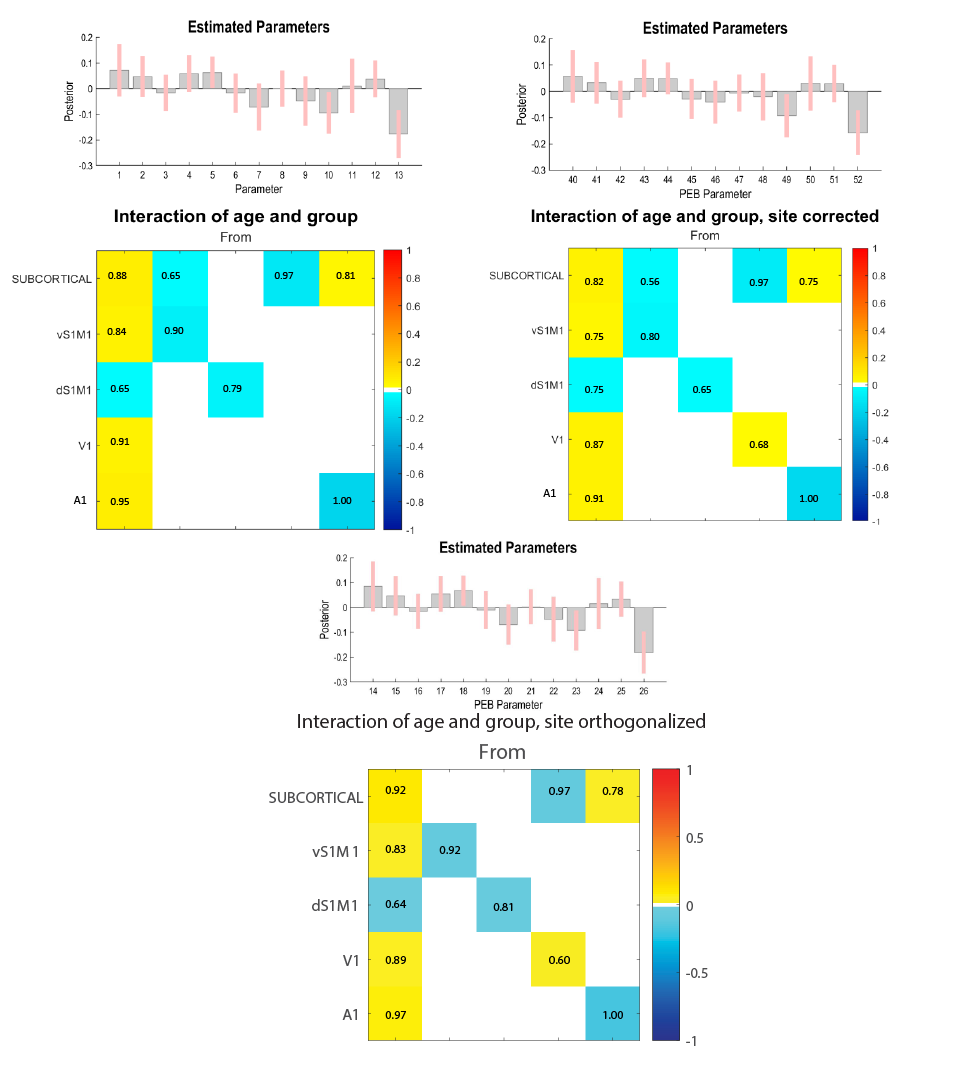


**Figure S10. Interaction between age and group.** SPM graphical output when displaying the influence of the interaction between age and group on DCM parameters. Top left: output of the analysis run without correcting for site; Top right : output of the analysis run correcting for site; Bottom row: output of the analysis run orthogonalizing site to age. For each result we show the barplot (*top*) showing effect sizes (gray bars) and 90% confidence intervals (pink lines), and the effective connectivity matrix (*bottom*) showing the effect of the interaction term on intrinsic connectivity for each region (values on the diagonal), bottom-up (values in the first column) and top-down (first row) connections. Colors show estimated effect in Hz while annotated numbers show posterior probabilities for each connection.

## Association with Social Responsiveness Scale

**Table S2. Linear mixed models investigating association of SRS, age and their interaction on estimated DCM parameter strengths.**

| *Connection* | *Contrast* | *Estimates* | *Confidence Interval* | *P-Value* | *P-Value (FDR corrected)* |
| --- | --- | --- | --- | --- | --- |
| vS1 self connection |  |  |  |  |  |
|  | Age | -0.07 | -0.46-0.33 | 0.74 |  |
|  | SRS | 0.001 | -0.05-0.05 | 0.69 |  |
|  | Age*SRS | 0.001 | -0.01-0.01 | 0.91 | 0.916 |
| A1 self connection |  |  |  |  |  |
|  | Age | 0.05 | -0.33–0.42 | 0.81 |  |
|  | SRS | -0.01 | -0.01-0.01 | 0.81 |  |
|  | Age*SRS | -0.01 | -0.01-0.01 | **0.013*** | **0.053*** |
| Subcortical to V1 |  |  |  |  |  |
|  | Age | 0.04 | -0.32-0.4 | 0.84 |  |
|  | SRS | -0.01 | -0.01-0.01 | 0.17 |  |
|  | Age*SRS | -0.01 | -0.01-0.01 | 0.71 | 0.916 |
| Subcortical to A1 |  |  |  |  |  |
|  | Age | -0.13 | -0.37-0.11 | 0.29 |  |
|  | SRS | -0.01 | -0.01-0.01 | 0.19 |  |
|  | Age*SRS | -0.01 | -0.01-0.01 | 0.52 | 0.916 |

To confirm the results found using linear models, and for consistency with the DCM framework, we repeated the SRS by age interaction analysis using the PEB pipeline described above. In this case, we modelled the influence of SRS, age and their interaction on DCM parameters. We added mean FD as a covariate to correct for the effect of motion and dummy variables for sites. The output of this analysis is in line with results of linear models and is shown in Figure S11.

#
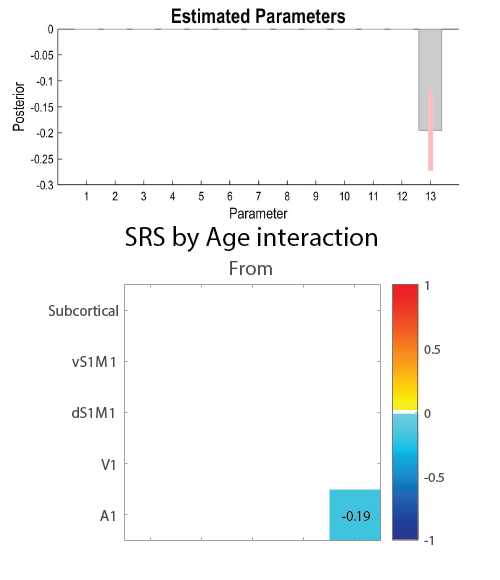


**Figure S11. Interaction between SRS and age.** SPM graphical output when displaying the influence of the interaction between age and SRS on DCM parameters. Barplot (*top*) showing effect sizes (gray bars) and 95% confidence intervals (pink lines), and the effective connectivity matrix (*bottom*) showing the effect of the interaction term on intrinsic connectivity for each region (values on the diagonal), bottom-up (values in the first column) and top-down (first row) connections. Colors show estimated effect in Hz while annotated numbers show posterior probabilities for each connection.

#

# Supplementary Discussion Notes

## Recent contributions from dynamic functional connectivity methods

Most of the functional connectivity studies we considered in our manuscript report results obtained with traditional functional connectivity, that is by assuming the stationarity of the connectivity pattern between regions over time (like in our analyses). Recent investigations in the dynamic reconfigurations of connectivity patterns (dynamic functional connectivity - dFC) might help to further describe the nature of functional segregation and integration in ASD. For instance, the previously reported functional hyperconnectivity (i.e. enhanced internal coherence of fMRI time courses) within the salience network in ASD [(Uddin et al. 2013)](https://paperpile.com/c/vtEbRx/IatvN) aligns with recent findings from dFC reporting a decreased permanence of subcomponents of this network in a coactivated state with both default-mode network (DMN) and central executive network (CEN) [(Marshall et al. 2020)](https://paperpile.com/c/vtEbRx/fGNwS). A similar result had been reported in a previous dFC study, however coexisting also with patterns of *hyperconnectivity* between the same networks. In that respect the authors proposed that these apparently divergent findings could actually reflect situations of both overconnectivity and underconnectivity across different dynamic states [(Rashid et al. 2018)](https://paperpile.com/c/vtEbRx/iZg7v). In general, beyond the traditional measure of overall static connectivity, dFC extracts different transient “states” - each one characterized by the interaction of a set of brain regions - and quantifies for each one the average amount of time spent in a given state (mean dwell time), and its ratio relative to the total time of fMRI acquisition. By employing these measures, recent studies showed in ASD either an overall increased segregation of the DMN and longer dwell times in a globally disconnected state [(Rashid et al. 2018)](https://paperpile.com/c/vtEbRx/iZg7v), or an increased “integration” (“overconnection”) of the DMN with CEN and fronto-parietal network [(Mash et al. 2019)](https://paperpile.com/c/vtEbRx/NM6I). In the latter study, the reduced segregation of the DMN was also accompanied by underconnectivity within the auditory network, and between fronto-parietal and visual network.

Although interesting, and revealing the complexity of connectivity information that can be extracted from fMRI data, these results can be challenging to interpret - as admittedly is the case for all fMRI-based functional/effective connectivity studies, including ours. While dFC provides a methodological approach to look in greater detail at connectivity than static functional connectivity, some necessary methodological choices can influence the results, such as the fact of defining regions of interest using ICA (see in this respect also the section “Choice of the region of interest: meta-ICA component vs. spherical ROI” above for our study) and identifying transient states using k-means clustering, as noted by the authors of one of these studies [(Mash et al. 2019)](https://paperpile.com/c/vtEbRx/NM6I). The same authors also note how the connection between dFC metrics and traditional measures of (static) overconnectivity/underconnectivity can be challenging, as for instance the presence of underconnectivity “does not necessarily indicate ‘weaker’ connections, but may instead reflect greater variability in FC over time”. These standing issues should be mentioned not to diminish the undeniable importance of investigating transient patterns of functional connectivity, but instead to stress the complexity of these novel measures, which unfortunately to date still do not translate into an effective tool to resolve the sometimes diverging results of the traditional static functional connectivity approach [(Muller et al. 2011; Khan et al. 2015)](https://paperpile.com/c/vtEbRx/2v95+UHgd).

# References

[Beckmann, C. F., and S. M. Smith. 2004. “Probabilistic Independent Component Analysis for Functional Magnetic Resonance Imaging.” *IEEE Transactions on Medical Imaging* 23 (2): 137–52.](http://paperpile.com/b/vtEbRx/NNXS)

[Cerliani, L., M. Mennes, R. M. Thomas, A. Di Martino, M. Thioux, and C. Keysers. 2015. “Increased Functional Connectivity Between Subcortical and Cortical Resting-State Networks in Autism Spectrum Disorder.” *JAMA Psychiatry*  72 (8): 767–77.](http://paperpile.com/b/vtEbRx/QDSs)

[Di Martino, A., C. G. Yan, Q. Li, E. Denio, F. X. Castellanos, K. Alaerts, J. S. Anderson, et al. 2013. “The Autism Brain Imaging Data Exchange: Towards a Large-Scale Evaluation of the Intrinsic Brain Architecture in Autism.” *Molecular Psychiatry* 19: 659–67.](http://paperpile.com/b/vtEbRx/XIaRk)

[Friston, Karl J., Joshua Kahan, Bharat Biswal, and Adeel Razi. 2014. “A DCM for Resting State fMRI.” *NeuroImage* 94 (July): 396–407.](http://paperpile.com/b/vtEbRx/j4LOf)

[Friston, Karl J., Vladimir Litvak, Ashwini Oswal, Adeel Razi, Klaas E. Stephan, Bernadette C. M. van Wijk, Gabriel Ziegler, and Peter Zeidman. 2016. “Bayesian Model Reduction and Empirical Bayes for Group (DCM) Studies.” *NeuroImage* 128 (March): 413–31.](http://paperpile.com/b/vtEbRx/49Cnn)

[Genovese, Christopher R., Nicole A. Lazar, and Thomas Nichols. 2002. “Thresholding of Statistical Maps in Functional Neuroimaging Using the False Discovery Rate.” *NeuroImage* 15 (4): 870–78.](http://paperpile.com/b/vtEbRx/1GjMn)

[Khan, Sheraz, Konstantinos Michmizos, Mark Tommerdahl, Santosh Ganesan, Manfred G. Kitzbichler, Manuel Zetino, Keri-Lee A. Garel, Martha R. Herbert, Matti S. Hämäläinen, and Tal Kenet. 2015. “Somatosensory Cortex Functional Connectivity Abnormalities in Autism Show Opposite Trends, Depending on Direction and Spatial Scale.” *Brain: A Journal of Neurology* 138 (Pt 5): 1394–1409.](http://paperpile.com/b/vtEbRx/UHgd)

[Marshall, Emily, Jason S. Nomi, Bryce Dirks, Celia Romero, Lauren Kupis, Catie Chang, and Lucina Q. Uddin. 2020. “Coactivation Pattern Analysis Reveals Altered Salience Network Dynamics in Children with Autism Spectrum Disorder.” *Network Neuroscience (Cambridge, Mass.)* 4 (4): 1219–34.](http://paperpile.com/b/vtEbRx/fGNwS)

[Mash, Lisa E., Annika C. Linke, Lindsay A. Olson, Inna Fishman, Thomas T. Liu, and Ralph-Axel Müller. 2019. “Transient States of Network Connectivity Are Atypical in Autism: A Dynamic Functional Connectivity Study.” *Human Brain Mapping* 40 (8): 2377–89.](http://paperpile.com/b/vtEbRx/NM6I)

[Mesulam, M. 2000. *Principles of Behavioural and Cognitive Neurology*. Oxford: Oxford University Press.](http://paperpile.com/b/vtEbRx/Vyizn)

[Muller, R. A., P. Shih, B. Keehn, J. R. Deyoe, K. M. Leyden, and D. K. Shukla. 2011. “Underconnected, but How? A Survey of Functional Connectivity MRI Studies in Autism Spectrum Disorders.” *Cerebral Cortex* . https://doi.org/](http://paperpile.com/b/vtEbRx/2v95)[10.1093/cercor/bhq296](http://dx.doi.org/10.1093/cercor/bhq296)[.](http://paperpile.com/b/vtEbRx/2v95)

[Nichols, T. E., and A. P. Holmes. 2002. “Nonparametric Permutation Tests for Functional Neuroimaging: A Primer with Examples.” *Human Brain Mapping* 15 (1): 1–25.](http://paperpile.com/b/vtEbRx/Qxr3y)

[Nickerson, Lisa D., Stephen M. Smith, Döst Öngür, and Christian F. Beckmann. 2017. “Using Dual Regression to Investigate Network Shape and Amplitude in Functional Connectivity Analyses.” *Frontiers in Neuroscience* 11 (March): 115.](http://paperpile.com/b/vtEbRx/44eR4)

[Rashid, Barnaly, Laura M. E. Blanken, Ryan L. Muetzel, Robyn Miller, Eswar Damaraju, Mohammad R. Arbabshirani, Erik B. Erhardt, et al. 2018. “Connectivity Dynamics in Typical Development and Its Relationship to Autistic Traits and Autism Spectrum Disorder.” *Human Brain Mapping* 39 (8): 3127–42.](http://paperpile.com/b/vtEbRx/iZg7v)

[Razi, Adeel, Joshua Kahan, Geraint Rees, and Karl J. Friston. 2015. “Construct Validation of a DCM for Resting State fMRI.” *NeuroImage* 106 (February): 1–14.](http://paperpile.com/b/vtEbRx/gnHTz)

[Uddin, Lucina Q., Kaustubh Supekar, Charles J. Lynch, Amirah Khouzam, Jennifer Phillips, Carl Feinstein, Srikanth Ryali, and Vinod Menon. 2013. “Salience Network–Based Classification and Prediction of Symptom Severity in Children With Autism.” *JAMA Psychiatry*  70 (8): 869–79.](http://paperpile.com/b/vtEbRx/IatvN)

[Zeidman, Peter, Amirhossein Jafarian, Nadège Corbin, Mohamed L. Seghier, Adeel Razi, Cathy J. Price, and Karl J. Friston. 2019. “A Guide to Group Effective Connectivity Analysis, Part 1: First Level Analysis with DCM for fMRI.” *NeuroImage* 200 (October): 174–90.](http://paperpile.com/b/vtEbRx/FBZac)

[Zhou, Yuan, Peter Zeidman, Shihao Wu, Adeel Razi, Cheng Chen, Liuqing Yang, Jilin Zou, Gaohua Wang, Huiling Wang, and Karl J. Friston. 2018. “Altered Intrinsic and Extrinsic Connectivity in Schizophrenia.” *NeuroImage. Clinical* 17: 704–16.](http://paperpile.com/b/vtEbRx/yCEHV)
